# Evaluation of milk sample fractions for characterization of milk microbiota from healthy and clinical mastitis cows

**DOI:** 10.1371/journal.pone.0193671

**Published:** 2018-03-21

**Authors:** Svetlana Ferreira Lima, Marcela Lucas de Souza Bicalho, Rodrigo Carvalho Bicalho

**Affiliations:** Department of Population Medicine and Diagnostic Sciences, Cornell University, Ithaca, New York, United States of America; University of Illinois, UNITED STATES

## Abstract

Amplicon sequencing technique has been increasingly applied to the clinical setting as a sensitive diagnostic tool. Therefore, it is of great importance to develop a DNA extraction method that accurate isolates DNA from complex host-associated microbiota. Given the multifactorial etiology of clinical mastitis and the diversified lifestyle of bacterial species harboring in milk, here four distinct milk sample fractions: raw whole milk, milk fat, casein-pellet, and casein-pellet + fat from healthy cows and cows with clinical mastitis, were subjected to bead-beating DNA extraction, followed by high-throughput sequencing. We aimed to identify the best approach for characterization of the milk microbiota and detection of mastitis pathogens (*Klebsiella* spp., *Streptococcus* spp. and *Escherichia coli*). DNA from each milk fraction tested was extracted by two commercial kits, which include physical, mechanical and chemical lysis; in total 280 DNA samples from 35 cows were analyzed. Milk-health-status were categorized into four groups (healthy group; *E*. *coli*-mastitis group; *Klebsiella* spp.-mastitis group; and *Streptococcus* spp.–mastitis group). Bacterial phyla and families were described for each milk-health-status group across milk sample fractions and DNA extraction kits. For the mastitis groups the relative abundance of f__Enterobacteriaceae and f__Streptococcaceae were compared to determine the efficacy of procedures in detecting the mastitis pathogens. The four milk fractions used allowed efficiently and uniformly detection of the causative agent of mastitis. Only 27% of the families detected in healthy milk were shared among the samples extracted from all fractions of milk samples; followed by 3, 4, and 12% for the samples from *E*. *coli*-mastitis, *Klebsiella* spp.-mastitis and *Streptococcus* spp-mastitis, respectively. However, the shared families comprised a mean relative abundance greater than 85%, regardless of milk-health-status, milk fraction and DNA isolation method. Taxonomic data at the family level showed that sequences from mastitis milk samples cultured positive for *E*. *coli* and *Klebsiella* spp. were predominantly affiliated with f__Enterobacteriaceae, while for *Streptococcus* spp. were dominated by f__Streptococcacea, followed by f__Pseudomonadaceae and f__Enterococcaceae. Microbial community analysis revealed that most of the microbial community composition corresponded to milk bacterial species irrespective of the DNA isolation method and milk fraction evaluated.

## Introduction

Inflammation of the mammary gland, also known as mastitis, is arguably the most important disease affecting dairy herds worldwide [1]. Mastitis is a complex disorder mainly triggered by bacterial infection [2, 3], typically by *Streptococcus* spp., *Staphylococcus aureus*, and non-aureus staphylococci, *Escherichia coli* and *Bacillus* spp., [4–6]. Due to its multifactorial etiology and the risk of antibiotic resistance, the best approach to mastitis treatment is to accurately identify the causative agent [7], which typically has been carried out by microbiological culture [6, 8], a standard diagnostic tool in veterinary medicine [9]. However, because cultures of mastitic milk samples may not always result in bacterial growth, an increasing number of studies has shown the potential of molecular techniques to improve the diagnosis of mastitis, with high sensitivity and specificity [10–13].

Accordingly, for any PCR-based approach, generating high-quality DNA is both critical and a challenge for accurate taxonomic profiling. Milk, in particular, is a challenging sample due to its physical and chemical characteristics, especially its fat, protein and, calcium constituents that act as PCR inhibitors [14]. Furthermore, clinical samples from an infected mammary gland contain additional PCR inhibitory factors such as bacterial and mammalian cellular debris [14], whereas non-clinical milk samples typically have low bacterial loads [15]. Likewise, different bacterial species may possess distinct cell-structural characteristics that may affect DNA recovery, thus the treatment applied to the sample could bias the results of the downstream analysis and consequently the taxonomic profiling.

Milk is a complex biological fluid mainly composed of fat globules and casein micelles (the primary group of milk proteins containing 80% of the total milk protein). All other proteins found in suspension in the fluid phase after precipitation of caseins are grouped together under the name of whey proteins [16]. In addition to the soluble non-casein proteins, the whey supernatant (milk serum) also contains water and lactose, and the two main components of the serum proteins in bovine milk are α lactalbumin and β lactoglobulin [17]. Typically, isolation of DNA from milk samples is performed by pelleting the casein [18–20]. Recently, Quigley et al. (2012) evaluated seven DNA isolation methods for raw milk and its derivate in terms of their relative success based on DNA yield and purity, as well as the quality of the template for downstream PCR. In the same study, DNA was isolated by resuspending the casein-pellet, which was submitted to different enzymatic and mechanical cell-lysis protocols [18]. However, some bacterial species have a diversified lifecycle in the milk environment, and the growth, location, and distribution of bacterial colonies in dairy products are important factors for the dairy food industry. For instance, starter, non-starter, spoilage, and pathogenic bacteria all become entrapped in the developing casein matrix of dairy foods [21]. On the other hand, recent studies have proposed an optimized milk template preparation for more efficient detection of *Mycobacterium avium* subsp. *paratuberculosis* by PCR, which involves combining the cream and pellet to produce a milk sample-template with increased PCR sensitivity [22, 23]. Furthermore, Staphylococci bacteria appear to bind to the fat globules and/or to the milk fat globule membrane [24, 25]; thus, PCR-based assays using traditional DNA isolation methodologies might affect the detection accuracy of certain bacterial species.

DNA extraction protocols that include bead-beating treatment have been resulted in a more accurate microbial DNA isolation than methods that do not include this treatment [26], therefore providing a more representative community of the original bacterial population. Additionally, bead-beating based kits have been recently applied to the isolation of DNA from healthy and mastitis milk samples [27, 28], and harsher milk samples such as colostrum [29]. Therefore, in the present study, DNA from four distinct milk sample fractions: raw whole milk, milk fat, casein-pellet, and casein-pellet and fat combined, were isolated by bead-beating treatment and subject to 16S amplicon sequencing in order to evaluate the impact of different milk fractions and extraction protocols on the microbiota composition of milk samples collected from healthy and mastitic quarters of dairy cows. Additionally, we aimed to identify the best DNA isolation method that allows us to accurately detect the main bacterial species that play a critical role in bacterial mammary infections (*E*. *coli*, *Klebsiella* spp. and *Streptococcus* spp.) in the U.S. dairies [30]. The DNA isolation methods were statistically evaluated according to the following criteria: amplicon concentration, protocol agreement, microbial representativeness, and reproducibility.

## Materials and methods

### Ethics statement

This study was conducted in one large commercial dairy farm situated in upstate New York due to its long-standing relationship with the Ambulatory Clinic at Cornell University. The research protocol was reviewed and approved by the Cornell University Institutional Animal Care and Use Committee (protocol number 2013–0056). The methods were carried out in accordance with the approved guidelines.

### Case definition

#### Clinical mastitis

Clinical mastitis examination was performed at the milking parlor by one of the veterinarians of the research team. Clinical mastitis was defined as the presence of visually abnormal milk (i.e. presence of flakes, clots, or serous milk) independently of systemic illness and signs of inflammation of the mammary gland during fore-stripping performed at the milking parlor.

#### Healthy cows

Cows with no visible changes of the secretion and/or the consistency of the mammary tissue were classified as healthy. Additionally, cows were not eligible to be included in the study if they were diagnosed with clinical mastitis in the current lactation and when antimicrobial or anti-inflammatory treatment occurred within the previous 30 d, when cows were within 5 d post-calving, within 30 d of drying off, with visible signs of teat damage, or experiencing concurrent disease.

### Sample collection

For culture and metagenomics analysis, milk samples were aseptically collected at the milk parlor before milking, at the morning milking, from a convenience sample of 54 Holstein dairy cows, of which 35 cows were diagnosed with clinical mastitis and 19 were healthy cows. Sampling methods followed standard recommendations by the National Mastitis Council’s Laboratory Handbook on Bovine Mastitis [6]. Briefly, the first streams of milk from each quarter were discarded for mammary gland stimulation, and subsequently the teats were dipped in iodine tincture. Then teats were cleaned and disinfected using 70% ethanol, the first three streams were discarded, and the milk samples were collected into sterile plastic tubes without preservative (Corning Life Sciences, Tewksbury, MA). Approximately 50 ml of milk were collected in a single sterile 50-ml centrifuge tube (Fisher Scientific, Pittsburgh, PA) from each study cow. Milk samples from cows with mastitis were collected from the mastitic quarter, and milk samples from healthy cows were collected at random from one of the cow’s hind quarters. Samples were kept on ice until transported to the laboratory, a 2-ml aliquot was separated for culture analysis and the remaining 48-ml sample was stored at -20°C for further processing.

### Microbiological culture

To identify suitable samples for downstream analysis that are representative of the bacterial community present in each milk sample, we used an agar-plate culture system for identification of the main milk pathogens associated with clinical mastitis or confirmation of pathogen-free milk. Milk samples from all cows used in this study were submitted to our laboratory at Cornell University, Ithaca, NY, for bacterial identification using a chromogenic culture system (Accumast®, FERA Animal Health LCC, Ithaca, NY). Using this approach, the expected microbial relative abundances from our study samples, which were further determined by 16S rRNA amplicon sequencing, would be directly associated to the presence of the predicted main pathogen. The choice for this agar-plate culture system was due to its proven suitability for use under field conditions and its substantial overall accuracy for detection of common mastitis pathogens, which was previously confirmed by 16S rRNA gene sequencing [31].

**Fig 1:**
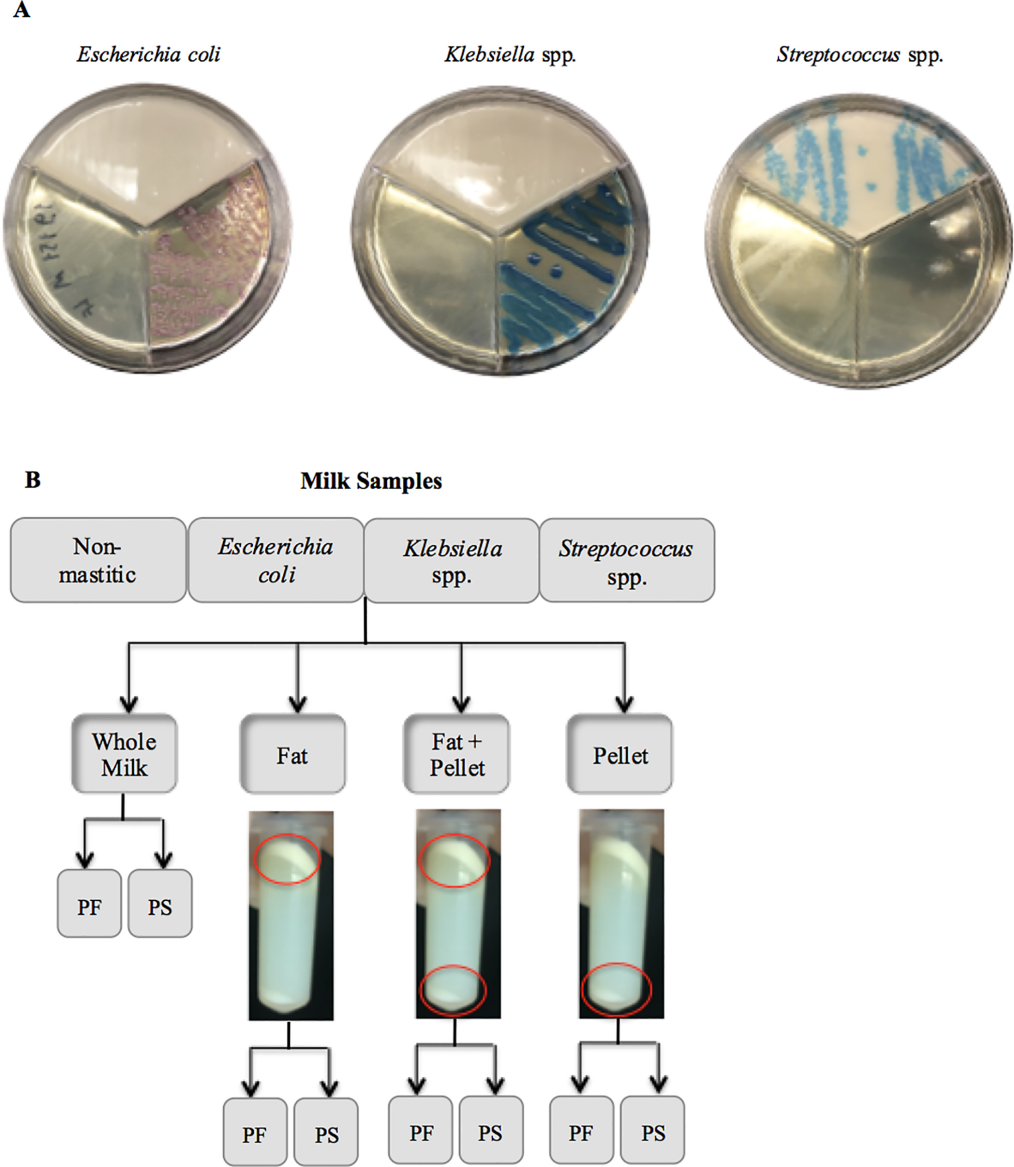
Visual assessment of *Escherichia coli*, *Klebsiella* spp. and *Streptococcus* spp. growth on Accumast plates performed in the laboratory (A). Overview of the experimental design (B). PF, PowerFood microbial DNA isolation kit; PS, PowerSoil microbial DNA isolation kit.

Milk samples were plated on the surface of each selective growth medium (tri-plate system) (Fig 1A) using sterile cotton swabs. Plates were aerobically incubated at 37°C for 24 h and subsequently read by one of the research team members. The threshold selected for considering a sample positive for bacterial growth using the Accumast culturing system was the presence of five or more colonies in a single section of the plate. Presence of bacterial growth in each of two different sections of the plate was considered a mixed infection and counted as positive for both types of bacteria. Classification of milk pathogens was performed following instructions of a flowchart developed by the manufacturer based on characteristics of growth of American Type Culture Collection (ATCC) strains as shown in Fig 1A.

Overall nineteen samples were excluded from the study because of (i) aerobic culture reported positive by milk samples from cows previously classified as healthy (n = 5 cows), (ii) mastitic-milk cultures that resulted in no-growth (n = 7 cows) or (iii) showed mixed pathogens (n = 7 cows). Therefore, milk samples from 35 cows (9 primiparous and 26 multiparous) were subjected to DNA extraction, of which, 14 samples originated from healthy cows, 4 samples from *E*. coli-mastitis cows, 4 from the *Klebsiella* spp.-mastitis and 13 samples from *Streptococcus* spp.-mastitis cows.

### Description of the DNA extraction kits

The detailed description of the two commercial kits used in the present study are described below.

PowerFood DNA isolation kit (MoBio, Qiagen Inc., Germantown, MD) is designed for “isolation of DNA from tough, food cultured microorganisms” and contains 0.15-mm garnet beads (PowerFood^®^ DNA Isolation Kit, MoBio, protocol). Microbial DNA isolation using the PowerFood kit is based on 6 steps: sample preparation (centrifugation of 1.8 ml of liquid food or homogenized 0.25 g of food in 0.75 ml of PBS, and then removal of the food residuals), collection of cells, cell lysis (samples are exposed to 65°C for 10 minutes followed by a bead beating process for 15 minutes, as suggested by the manufacturer), inhibitor removal, binding of DNA, wash procedure (removes residual salt and other contaminants), lastly an elution process. As a result, a final volume of 100 μl of the isolated DNA is collected in the final elution step.

PowerSoil DNA isolation kit (MoBio, Qiagen Inc., Germantown, MD) is designed for “isolation of environmental samples, including difficult soil types such as compost, sediment and manure” and contains 0.7-mm garnet beads. Microbial DNA isolation using this kit is based on 6 steps, similar to the steps performed in the PowerFood kit. However, in the cell lysis step, samples are exposed to a higher temperature of 70°C for 10 minutes followed by a bead beating process for 15 minutes, as suggested by the manufacturer. Additionally, in the wash step, samples are subjected to a more “intense” clean-up process to improve the purity of the final DNA template.

Reagent controls were tested for all DNA extraction kits used and subjected to PCR assay to prevent potential contamination attributed to reagent impurity. Negative results for the reagents and PCR controls were obtained according to agarose gel analysis. The DNA concentration of milk samples and control samples was evaluated by optical density using a NanoDrop ND-1000 spectrophotometer (NanoDrop Technologies, Rockland, DE) at wavelengths of 260 and 280 nm.

### Description of the milk sample fractions

DNA was isolated from four distinct milk sample fractions: whole milk, fat only, pellet only, and fat and pellet combined, as shown in Fig 1B. Frozen samples were thawed on the day of DNA extraction, homogenized and aliquoted in 7 Falcon tubes (15-ml volume) (Fisher Scientific, Pittsburgh, PA) each containing 6 ml of sample. The samples were then subjected to two different microbial DNA extraction kits (PowerFood and PowerSoil) for comprehensive microbial community analysis based on 16S rRNA gene sequencing. Samples from the same cow were processed at once, and then subjected to the same PCR assay, purification assay and sequencing batch. Only one member of our research team executed the DNA laboratory procedures, thereby circumventing inter-operator variability.

#### DNA obtained from whole milk

A total of 250 μl of milk from a Falcon tube containing 6ml-milk aliquot was transferred to a PowerSoil bead tube and DNA extraction was performed according to the manufacturer’s instructions. A total of 250 μl of milk from the same Falcon tube was transferred to a PowerFood bead tube, then mixed with 450 μl of Solution PF1 and transferred to a PowerFood microbead tube. DNA was then extracted according to the manufacturer’s instructions.

#### DNA obtained from milk fat

Two Falcon tubes (15-ml volume) each containing 6 ml of milk were used in this procedure. Milk samples were then transferred to a sterile 2 ml microcentrifuge tube (Eppendorf, Hauppauge, NY) and centrifuged at 16,000 × g for 5 minutes at 4°C. This process was repeated 3 times.

A total of 250 mg of fat content from tube 1 was transferred to a PowerSoil bead tube and DNA extraction was performed according to the manufacturer’s instructions. A total of 250 mg of fat content from tube 2 was resuspended in 450 μl of a strong lysing reagent (Solution PF1) from the PowerFood kit, further transferred to a PowerFood microbead tube, followed by DNA extraction according to the manufacturer’s instructions.

#### DNA obtained from milk casein-pellet

Two Falcon tubes (15-ml volume) each containing 6 ml of milk were used in this procedure. Milk samples were then transferred to two 2 ml microcentrifuge tubes, centrifuged at 16,000 × g for 5 minutes at 4°C and the supernatant composed of fat and whey was discarded from tube 1 (this process was repeated 3 times). The remaining pellet in tube 1 was washed with PBS two times and resuspended in 250 μl of the buffer solution used in the PowerSoil bead tubes and transferred to a PowerSoil bead tube. Similarly, supernatant composed of fat and whey was discarded from tube 2 (this process was repeated 3 times). The remaining pellet in tube 2 was washed with PBS two times and resuspended in 450 μl of Solution PF1 from the PowerFood kit and then transferred to a PowerFood microbead tube. DNA was then extracted according to the manufacturer’s instructions.

#### DNA obtained from milk fat + casein-pellet combined

Two Falcon tubes (15-ml volume) each containing 6 ml of milk were used in this procedure. Milk samples were then transferred to two 2 ml microcentrifuge tube and centrifuged at 16,000 × g for 5 minutes at 4°C. After centrifugation, the whey fraction was removed and discarded from tubes 1 and 2 (this procedure was repeated 3 times); fat and pellet contents were homogenized in 0.75 ml of UltraPure^TM^ distilled water, DNAse- and RNAse-free (Invitrogen Life Science Technologies, Grand Island, NY) for 5 minutes using a vortex with a horizontal adapter.

A total of 250 μl of fat + pellet content from tube 1 was transferred to a PowerSoil bead tube and another 250 mg from tube 2 were mixed in a sterile microcentrifuge tube containing 450 μl of Solution PF1 from the PowerFood kit and then transferred to a PowerFood microbead tube. DNA was then extracted according to the manufacturer’s instructions.

### PCR amplification of the bacterial 16S rRNA gene and amplicon sequencing

Amplification of the V4 hypervariable region from the 16S rRNA gene was performed by PCR using barcoded primers. Primers 515F and 806R were used according to previously described methods and optimized for the Illumina MiSeq platform [32]. In total, 280 different 12-bp error-correcting Golay barcodes primers were designed based on “The Earth Microbiome Project” (http://www.earthmicrobiome.org/) [33]. The PCR were performed using 10 μM of each primer (515F and 806R), EconoTaq Plus Green 1× Master Mix (Lucigen, Middleton, WI), 5 to 50 ng of individual metagenomic DNA samples, and ultrapure water to bring the final reaction volume to 25 μL. Blank controls in which no DNA was added to the reaction were also performed. All reactions were set up in triplicate, and the PCR conditions for amplification included an initial denaturing step of 94°C for 3 min followed by 35 cycles of 94°C for 45 s, 50°C for 1 min and 72°C for 90 s and a final elongation step of 72°C for 10 min (http://press.igsb.anl.gov/earthmicrobiome/protocols-and-standards/16s/). Replicate amplicons were pooled and purified using a Gel PCR DNA Fragment Extraction kit (IBI Scientific, Peosta, IA) and visualized by electrophoresis through 1.2% (wt/vol) agarose gels stained with 0.5 mg/ml ethidium bromide.

Samples that failed to be detected by PCR assay (no bands on the agarose gel) were re-tested; thus, a new PCR and agarose gel procedure were performed for confirmation of the negative result or for recovering of the false-negative sample. Concentrations from the resulting amplicons that were generally detected by gel electrophoresis were evaluated by optical density using a NanoDrop ND-1000 spectrophotometer (NanoDrop Technologies, Rockland, DE) at wavelengths of 260 and 280 nm.

Aliquots of milk amplicon samples were diluted to the same concentration and then pooled into one unique run according to individual barcode primers for the 16S rRNA gene, V4 hypervariable region. Final equimolar libraries were sequenced using the MiSeq reagent kit v2 (300 cycles) on the MiSeq platform (Illumina Inc., San Diego, CA).

### Bioinformatics

The 16S rRNA gene sequences generated were processed through the open source software pipeline Quantitative Insights Into Microbial Ecology 2 (QIIME 2) version 2017.2 (http://qiime2.org). Sequences were demultiplexed using the “demux emp-single” command. Quality control was performed using DADA2 [34] by removing any remaining phiX reads, chimeric sequences and low-quality regions of the sequences [34]. Herein, high-quality bases equal to Q30 (probability of an incorrect base call is 1 in 1000 and the inferred base call accuracy is 99.9%) were observed around position 150 bases, thus sequences were truncated at 150 bases. Dereplication was then performed by DADA2, which combines identical reads into “unique sequences” (the number of reads with that unique sequence), resulting in a higher-resolution amplicon variant table, which is analogue to the traditional Operational Taxonomic Table (OTU) [34]. The training feature “q2-feature-classifier” command using the Greengenes reference database [35] was created to classify representative sequences from our dataset. The output of this workflow is a classification of reads at multiple taxonomic levels: kingdom (k), phylum (p), class (c), order (o), family (f), genus (g) and species (s). Normalization of the OTU table for further downstream analysis was performed in QIIME 2 using “feature-table rarefy” command.

Alpha diversity, represented by Shannon index and Faith's Phylogenetic Diversity vector (Faith’ PD vector), was generated using QIIME 2 pipeline. Before calculation of Shannon and Faith’ PD vector samples were rarefied to an equal depth of 10,000 sequences. The same sequence depth used for alpha diversity computation was applied to beta diversity calculation (Weighted UniFrac). Principal coordinates were computed from the calculated UniFrac distance matrix to compress dimensionality into three-dimensional principal coordinate analysis (PCoA) plots created by QIIME 2 and visualized by EMPeror [36]. Differences between microbial communities (beta diversity) based on phylogenetic information visualized on the PCoA plots were calculated by permutational multivariate analysis of variance (PERMANOVA) in QIIME 2.

### Statistical analysis & sample-group categorization

To facilitate data analysis and interpretation of the results whole milk, fat, fat + pellet, and pellet were categorized as milk sample fractions. Additionally, milk-health-status groups were categorized into four groups: healthy group (consisted of samples collected from cows with no signs of clinical mastitis and showed negative culture results); and three mastitis groups (which consisted of samples collected from cows diagnosed with clinical mastitis): *E*. *coli*-mastitis group, *Klebsiella* spp.-mastitis group and *Streptococcus* spp.–mastitis group. Mastitis groups distinction was performed according to the main pathogen identified, *E*. *coli*, *Klebsiella* spp., and *Streptococcus* spp through standard culture methods.

The Pearson Chi-square test was performed using JMP Pro 12 (SAS Institute Inc.) to evaluate whether the number of samples positive in the PCR test differed between milk sample types (whole milk, fat, fat + pellet, and pellet) and DNA extraction kits (PowerFood and PowerSoil).

Differences in amplicon concentration, total number of sequences, total number of OTUs and alpha diversity (Shannon index and Faith’ PD vector) between milk sample fractions within or between DNA extraction kits were evaluated using the Mixed procedure in SAS 9.4 (SAS Institute Inc.) with Bonferroni to adjust for multiple comparisons.

The OTU data obtained from bioinformatics analysis were used to describe the relative abundances of bacterial phyla and families within each milk-health status group (*Escherichia coli* group, *Klebsiella* spp. group, *Streptococcus* spp. group, and healthy group) across all milk sample fractions and DNA extraction kits. Each value obtained indicated the percentage relative frequency of reads with 16S rRNA genes annotated to the indicated taxonomic level. The microbiota profile within milk groups was described for the most prevalent phyla and bacterial families using the tabulate function of JMP Pro 12. Graphs representing phyla mean relative abundance were constructed in Excel (Microsoft Corp., Redmond, WA), whereas a Heatmap was generated in JMP Pro 12 to graphically represent the relative distributions of the most common bacterial families found in our samples.

To gain a deeper insight into the dissimilarity levels of milk bacterial communities represented among samples extracted by the different DNA isolation methods (4 milk fractions × 2 DNA isolation kits), Venn diagrams (VennDiagram package under RStudio software version 0.99.903; RStudio, Inc) were created for graphical descriptions of the number of unique and shared bacterial families. Tables depicting the unique and shared families (core microbiome), and their respective relative abundances among DNA sample fractions were generated using the tabulate function of JMP Pro 12. Statistical analysis using Kruskal-Wallis test with Benjamin-Hochberg false discovery rate correction was applied to these data sets to derive statistically significant differences.

To test for differential abundance of taxa (at the family level) that might be driven by the different DNA isolation methods, Kruskal-Wallis tests followed by Benjamini-Hochberg false discovery rate (FDR) calculations were performed using JMP Pro 12. For the mastitic milk groups (*Escherichia coli*, *Klebsiella* spp., *Streptococcus* spp. groups) the relative abundances of f__Enterobacteriaceae (bacterial family known to comprise the *Escherichia coli* and *Klebsiella* spp. groups) and f__Streptococcaceae (bacterial family known to comprise the *Streptococcus* spp. group), were compared between the DNA isolation methods to determine the efficacy of the procedures in detecting the causative agent of mastitis. The graphs representing family mean relative abundances (MRA) were constructed in Excel (Microsoft Corp., Redmond, WA) and differences with a value of *P* ≤ 0.05 were considered significant.

## Results

### Descriptive statistics

In total, thirty-five samples (n = 35 cows) were subjected to DNA extraction and PCR screening. Equal volumes of each of the 35 milk samples were aliquoted in eight microcentrifuge tubes, which were processed as whole milk, fat, fat + pellet, and pellet by two different DNA extraction kits (PowerFood or PowerSoil), giving a total of 280 study samples. Of these 280 samples, 112 originated from healthy group, 32 from *E*. *coli-*mastitis group, 32 from *Klebsiella* spp.-mastitis group and 104 from *Streptococcus* spp.-mastitis group. Forty-nine samples (17.5% of total samples: healthy group = 14.3% and mastitis groups = 3.2%) were excluded because of no visible bands shown in the agarose gel. In total, 215 samples were subjected to amplicon sequencing and further evaluation.

Total post-quality-control number of sequences (sequences were filtered for size, quality, phiX reads, and for the presence of chimeras) used in the study was 15,177,739. The average coverage of sequences per sample was 68,061 (median = 57,844 sequences) with a standard deviation (SD) of 38,261.

### Amplicon concentration

To assure the quality of isolated nucleic acid, the samples were used for different downstream applications including PCR amplification and sequencing of PCR products.

Whole milk yielded significantly lower amplicon concentrations compared to samples from fat, fat + pellet, and pellet only (*P*-value < 0.0001, Table 1). Amplicon concentrations within *Klebsiella* spp.-mastitis samples were higher when fat was used as a milk sample type and benefited by PowerFood extraction kit (*P*-value < 0.025, Table 1). On the other hand, amplicon concentrations within *Streptococcus* spp.-mastitis samples were higher when pellet only was used as a milk sample type and benefited by PowerSoil extraction kit (*P*-value < 0.002, Table 1).

**Table 1:** Comparison of DNA and amplicon concentration among DNA extraction protocols using different milk sample fractions (whole milk, fat, fat + pellet, and pellet) according to milk-health status groups (healthy or mastitis caused by *Escherichia coli*, *Klebsiella* spp., *Streptococcus* spp., and all groups combined) and two different DNA extraction kits. Numbers in parentheses represent the standard error of the mean.

	Amplicon (ng/μl)				
Milk-health status	WM^1^	Fat	F+P^2^	Pellet	*P*-value
**PowerFood**					
Healthy	16.8 (6.3)	25.5 (6.6)	25.8 (5.7)	37.3 (5.4)	0.11
*Escherichia coli*	31.6 (13.4)	29.6 (13.4)	51.9 (13.4)	44.7 (15.5)	0.70
*Klebsiella* spp.	**63.3 (5.0)**^**a**^	**82.9 (5.0)**^**b**^	**62.3 (5.0)**^**a**^	**78.4 (5.0)**^**ab**^	**0.025**
*Streptococcus* spp.	41.6 (5.0)	55.2 (5.0)	47.2 (5.2)	52.8 (5.2)	0.24
Total	38.7 (3.2)	48.9 (3.4)	46.5 (3.2)	54.2 (3.3)	0.30
**PowerSoil**					
Healthy	11.5 (5.6)	13.7 (6.0)	23.0 (5.3)	29.8 (5.1)	0.07
*Escherichia coli*	22.6 (11.0)	36.3 (11.0)	37.2 (11.0)	38.8 (11.0)	0.71
*Klebsiella* spp.	47.5 (6.7)	65.1 (6.7)	70.9 (6.7)	70.3 (7.7)	0.10
*Streptococcus* spp.	**25.0 (4.6)**^**a**^	**37.2 (4.6)**^**ab**^	**48.6 (5.2)**^**b**^	**49.5.9 (4.7)**^**b**^	**0.002**
Total	**27.1 (3.3)**^**a**^	**37.2 (3.3)**^**ab**^	**46.0 (3.4)**^**b**^	**46.4 (3.3)**^**b**^	**0.001**
**All groups combined**	**33.0 (2.4)**^**a**^	**42.9 (2.4)**^**b**^	**46.3 (2.4)**^**b**^	**50.4 (2.4)**^**b**^	**<0.0001**

^a,b,c^ Different superscripts between values indicate a significant difference.

WM^1^: Whole Milk

F+P^2^: Fat + Pellet

### Number of sequences and number of OTUs

Although differences in amplicon concentrations were detected among milk sample fractions and extraction kits tested, sufficient amplicon yields from samples positive in the PCR assay (visible and appropriate size band in the agarose gel) were recovered for amplicon sequencing. The post-sequencing data after quality analysis and removal of low-quality sequences are described in Table A in S1 File. No significant differences between milk sample fractions and DNA extraction kits were observed in the number of 16S rRNA sequences and OTUs generated from amplicons of healthy and mastitis milk samples (*P*-value = 0.77, and *P*-value = 0.86, respectively, Table A in S1 File).

### Taxonomic profile at the phylum level

The on-farm culture system for bacterial identification was used as a criterion to assess the effectiveness of the DNA extraction methods on isolation of the DNA from milk microbial communities. Here, we evaluated how the taxa detection frequency was affected by different DNA isolation procedures.

**Fig 2:**
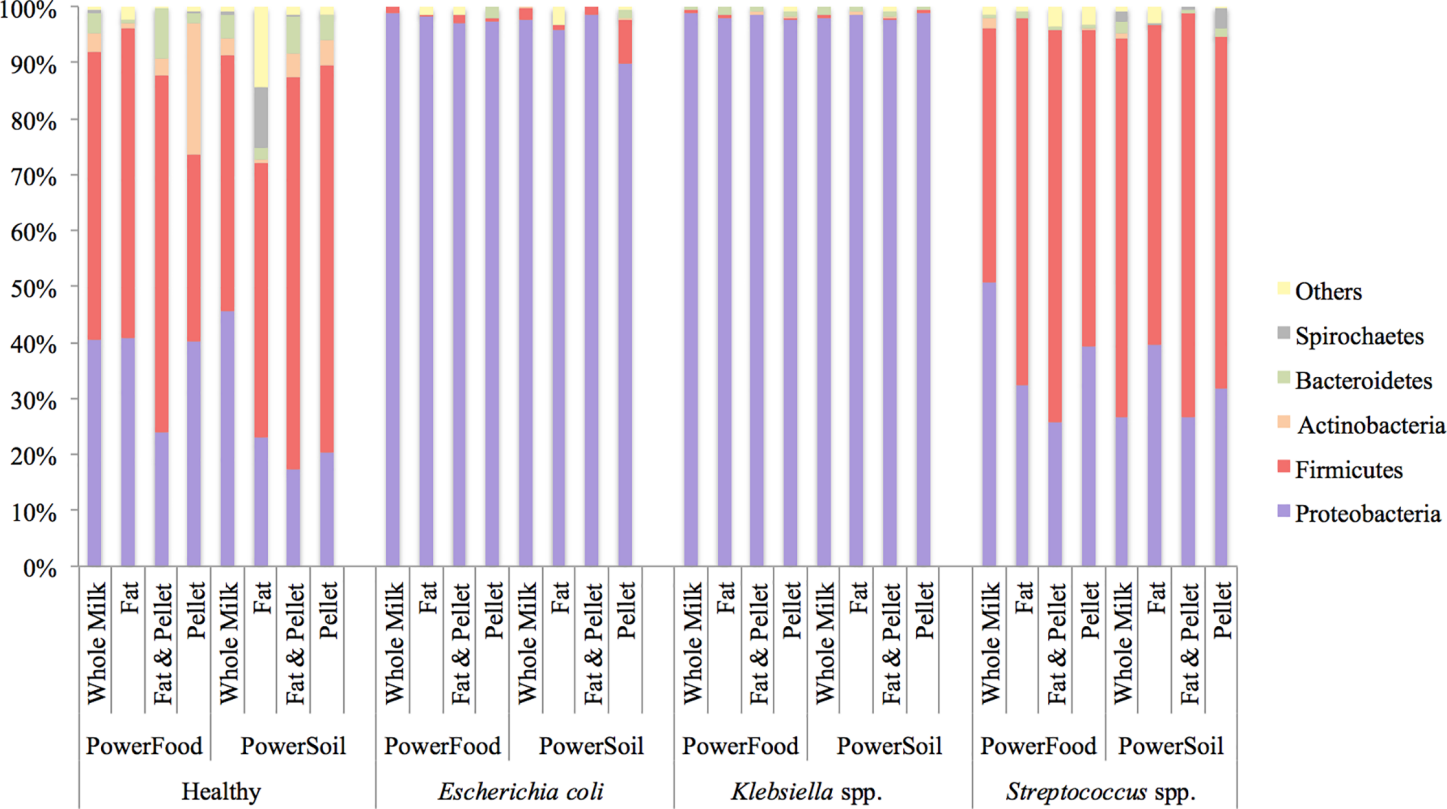
Mean relative abundance of the most prevalent bacterial phyla identified in healthy milk samples and milk samples from cows diagnosed with clinical mastitis due to *Escherichia coli*, *Klebsiella* spp. and *Streptococcus* spp. infection according to four milk sample fractions (whole milk, fat, fat + pellet, and pellet) and two different DNA extraction kits (PowerFood and PowerSoil).

The relative distribution of the most common phyla detected in healthy and mastitis milk samples are presented in Fig 2. Sequences affiliated with Firmicutes (MRA: 57.7%, standard error: ± 7.6) and Proteobacteria (MRA: 26.0 ± 7.6) dominated the healthy milk samples. No significant differences, with respect to the MRA of these two phyla, were observed when milk sample fractions and kits were compared (Figures A1 and A2 in S1 File, respectively). However, in *E*. *coli*-mastitis and *Klebsiella* spp.-mastitis, samples, the phylum Proteobacteria accounted for approximately 98% of the detected 16S rRNA sequences regardless of milk fractions and DNA extraction kits (Fig 2). In *Streptococcus* spp-mastitis samples, the majority of the sequences were affiliated with Firmicutes (MRA: 69.6 ± 9.5) and Proteobacteria (MRA: 30.1 ± 9.4, Fig 2).

### Taxonomic profile at the family level

#### Healthy milk

**Fig 3:**
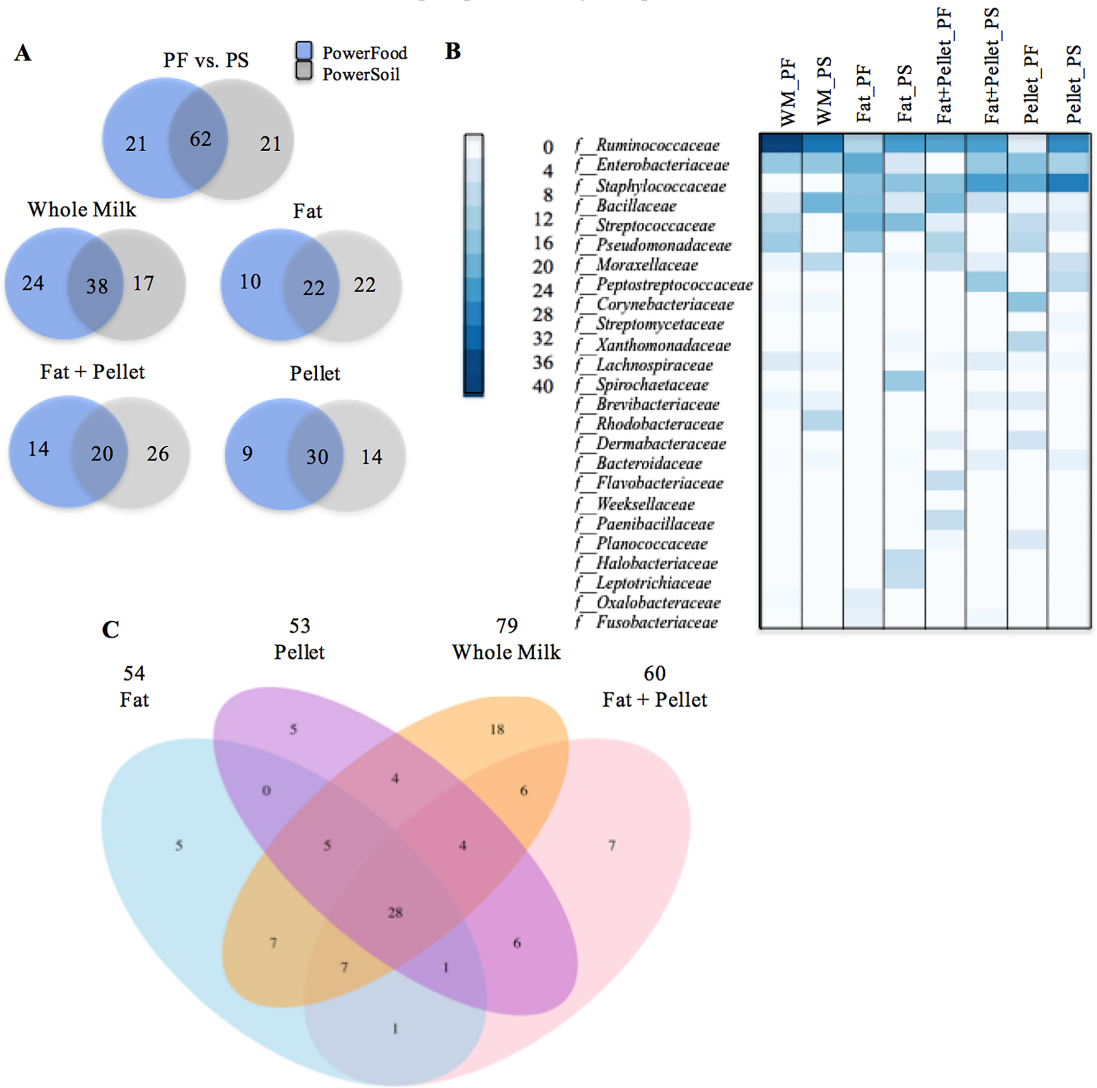
Venn diagrams showing the numbers of unique and shared bacterial families for healthy milk samples (A). Heatmap illustrating the 25 most common bacterial families ranking by relative abundance identified in healthy milk samples according to milk sample fractions: fat, fat + pellet, pellet, and whole milk (WM) and DNA extraction kit (PS and PF) (B). Venn diagram showing the numbers of unique and shared bacterial OTUs according to milk sample fractions. Numbers at the top of each milk sample type are the total number of families detected in samples processed by that protocol (C).

Fig 3A displays a Venn diagram illustrating the degree of overlap of bacterial families between the two DNA extraction kits for healthy milk samples. The Venn diagram shows that, overall, 62 families were shared between the samples extracted by the two DNA extraction kits. These 62 families combined held a MRA of 95.65% (± 1.56). Additionally, when comparison was made within milk sample fractions the number of shared bacterial families decreased to 38, 22, 20 and 30 for whole milk, fat, fat + pellet, and pellet, respectively. The most common bacteria families detected in healthy milk samples were represented by f__Ruminococaceae, f__Enterobacteriaceae, f__Staphylococcaceae, f__Bacillaceae, f__Streptococcaceae, and f__Pseudomonadaceae (Fig 3B).

The degree of overlap of bacterial families among the four milk sample fractions is described in Fig 3C. Twenty-eight shared families comprise the core microbiota identified among all four milk sample fractions. The core family held a MRA of 87.8% (± 2.4). A detailed description of the 28 shared families is given in Table 2 in S1 File. f__Ruminococaceae, f__Enterobacteriaceae, f__Bacillaceae, and f__Pseudomonadaceae were the top four bacterial families identified in milk samples regardless of the milk sample fractions (Table B in S1 File). The description of the types of unique families detected in each milk sample type and their respective relative abundances are given in Table C in S1 File.

#### *Escherichia coli*—mastitis milk

**Fig 4:**
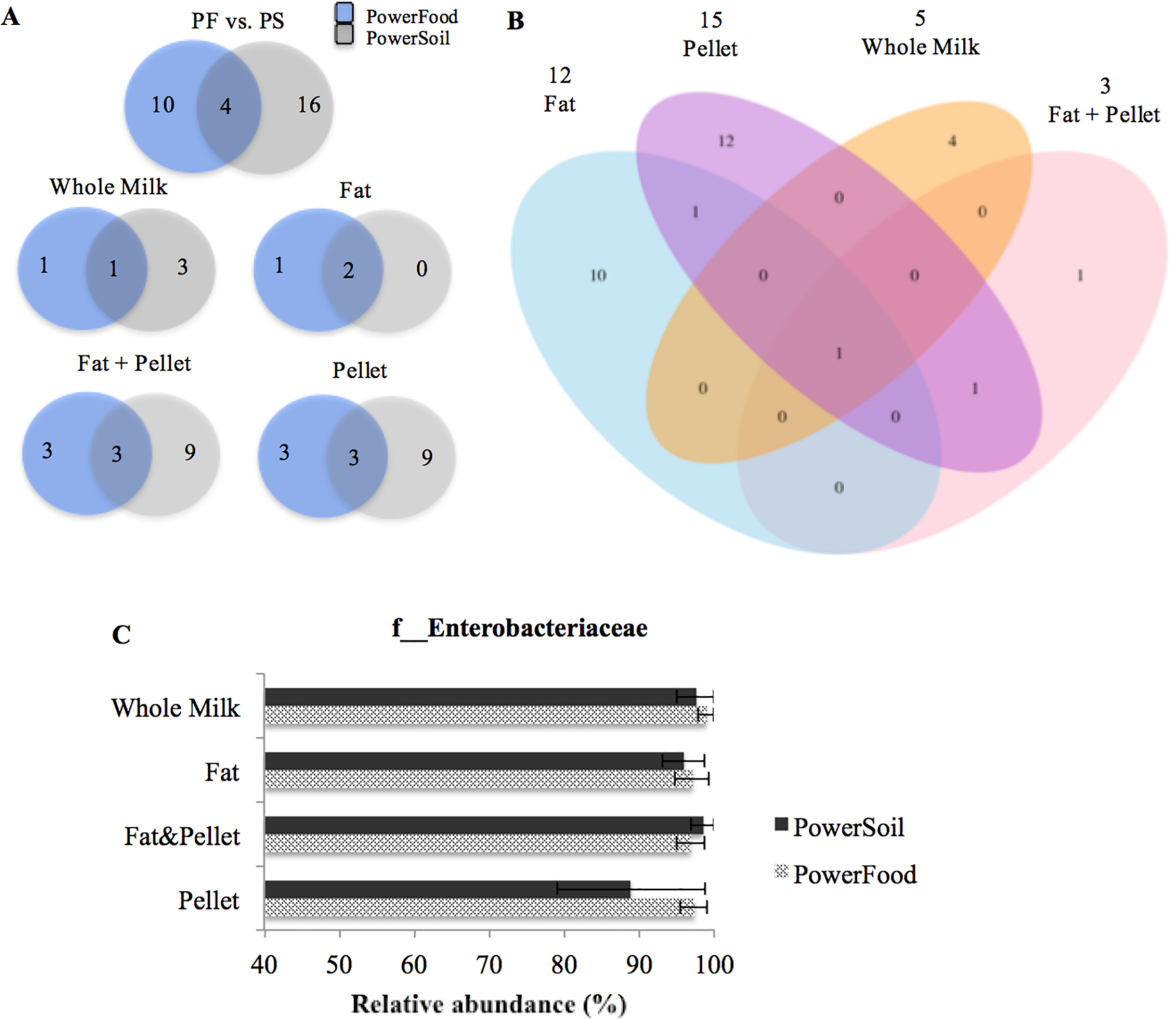
Venn diagram showing the numbers of unique and shared bacterial families for *Escherichia coli*-mastitis milk samples according to milk sample fractions: fat, fat + pellet, pellet, and whole milk (WM) and DNA extraction kit (PS and PF) (B). Numbers at the top of the milk sample type name indicate the total number of families detected in samples processed by that protocol. Mean relative abundance (MRA) of f__Enterobacteriaceae taxon detected in each milk sample fractions (C). Error bars represent the standard error of the mean.

Fig 4A shows a Venn diagram illustrating the degree of overlap of bacterial families between the two DNA extraction kits for *E*. *coli*-mastitis milk. Of the 30 families detected, only four were shared between the samples extracted by PowerFood and PowerSoil (Fig 4A). The core microbiota framework identified within both DNA extraction kits comprised f__Bacteroidaceae (with a MRA of 0.31% ± 0.31 and 0.31% ± 0.31), f__Ruminococcaceae (0.10% ± 0.10; and 0.50% ± 0.41), f__Staphylococcaceae (0.57%, ± 0.45; and 0.57% ± 0.39), and f__Enterobacteriaceae (97.51% ±0.8; and 94.77% ± 2.88) for PowerFood and PowerSoil, respectively. As displayed in Fig 4B, only one family, f__Enterobacteriaceae, comprised the core microbiota in the samples from whole milk, fat, fat + pellet, and pellet. In total, 10, 12, 4 and 1 bacterial families were exclusively found in samples extracted from fat, pellet, whole milk, and fat + pellet, respectively (Fig 4B). Details of the unique families detected in each milk sample fractions and their respective relative abundances are shown in Table D in S1 File.

We also evaluated whether the relative abundance of the mastitis causative agent identified by bacterial culture varied with the milk sample fraction and extraction kit. Fig 4C shows that the MRA for all OTUs affiliated to f__Enterobacteriaceae showed good reproducibility (MRA > 90%) across all milk sample fraction and both DNA extraction kits (Fig 4C). All DNA isolation protocols accurately detected f__Enterobacteriaceae, and no significant differences in the relative abundance of this taxon were observed among the DNA isolation protocols (*P*–value > 0.05, Fig 4C).

#### *Klebsiella* spp.- mastitis milk

**Fig 5:**
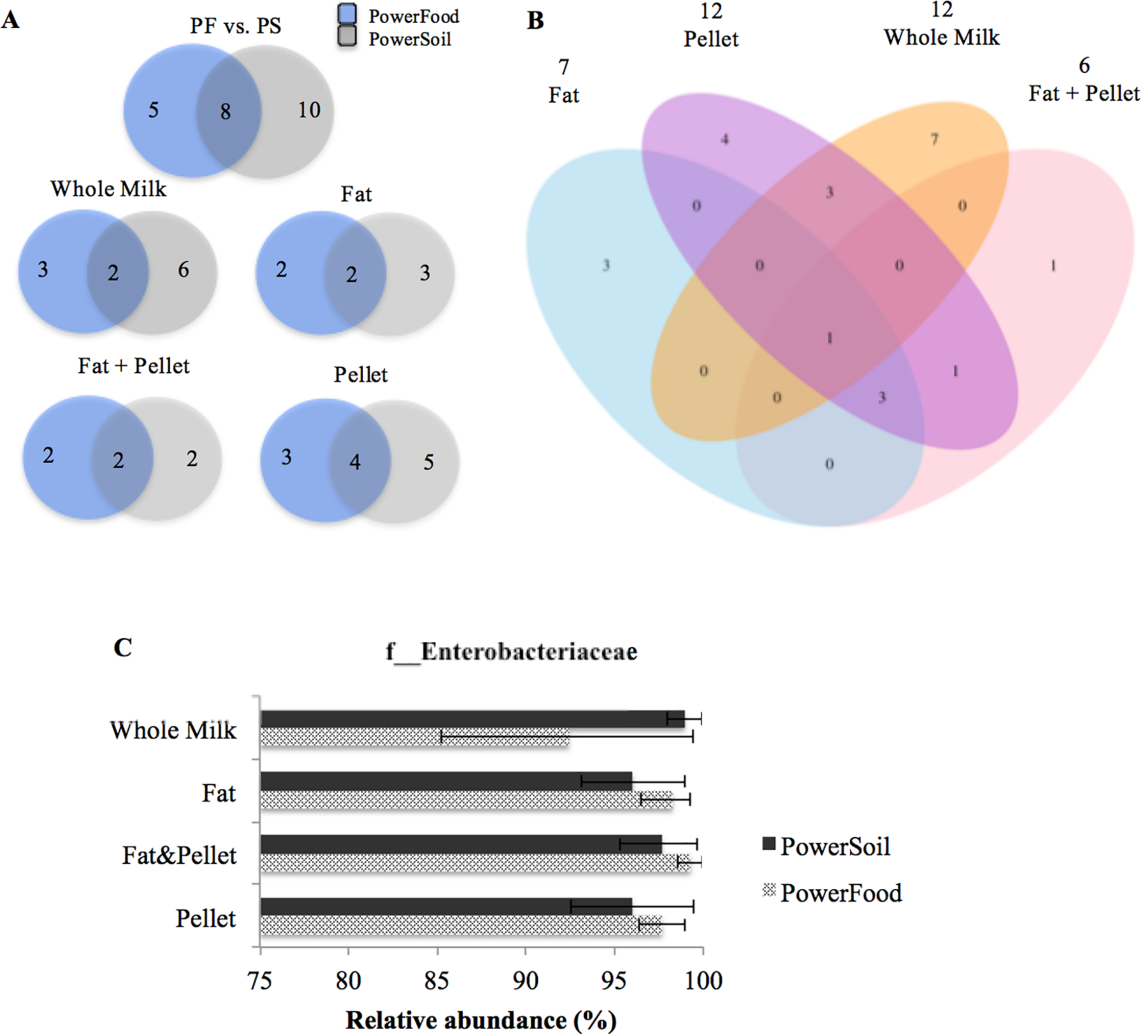
Venn diagram showing the numbers of unique and shared bacterial families for *Klebsiella* spp.-mastitis milk samples according to milk sample fractions: fat, fat + pellet, pellet, and whole milk (WM) and DNA extraction kit (PS and PF) (B). Numbers at the top of the milk sample type name indicate the total number of families detected in samples processed by that protocol. Mean relative abundance (MRA) of f__Enterobacteriaceae taxon in each milk sample fractions (C). Error bars represent the standard error of the mean.

Fig 5A shows a Venn diagram illustrating the degree of overlap of bacterial family between the two DNA extraction kits for *Klebsiella* spp-mastitis milk. Of the 28 families detected, only eight, f__Halobacteriaceae, f__Corynebacteriaceae, f__Ruminococcaceae, f__Brevibacteriaceae, f__Bacteroidaceae, f__Flavobacteriaceae, f__Pseudomonadaceae, and f__Enterobacteriaceae, were shared between the samples extracted by PowerFood and PowerSoil (Fig 5A). Their respective mean relative abundances within each DNA extraction kits (PowerFood and PowerSoil respectively) are described as follows: f__Halobacteriaceae (0.10 ± 0.10; and 0.30 ± 0.30;), f__Corynebacteriaceae (0.11 ± 0.11; and 0.23% ± 0.17), f__Ruminococcaceae (0.05%, ± 0.05; and 0.18% ±0.31), f__Bacteroidaceae (0.04%,± 0.03; and 0.06% ± 0.06), f__Flavobacteriaceae (0.55% ± 0.4; and 1.08% ± 0.62), f__Pseudomonadaceae (0.19% ± 0.14; and 0.61% ± 0.33), and f__Enterobacteriaceae (96.90% ± 1.82; and 97.16% ± 1.20). Whilst only one family, f__Enterobacteriaceae, was shared among the samples processed from whole milk, fat, fat + pellet, and pellet (Fig 5B). In total, 3, 4, 7, and 1 bacterial families were found to be unique to the samples extracted from fat, pellet, whole milk, and fat + pellet, respectively (Fig 5B). A more detailed description of the unique families detected in each protocol and their respective relative abundances are given in Table E in S1 File.

Fig 5C shows that the MRA for all OTUs affiliated to f__Enterobacteriaceae showed good reproducibility (MRA > 90%) across all milk sample fractions and DNA extraction kits. All DNA isolation protocols accurately detected f__Enterobacteriaceae, and no significant differences in the relative abundance of this taxon were observed among the DNA isolation procedures (*P*—value > 0.05, Fig 5C).

#### *Streptococcus* spp.- mastitis milk

**Fig 6:**
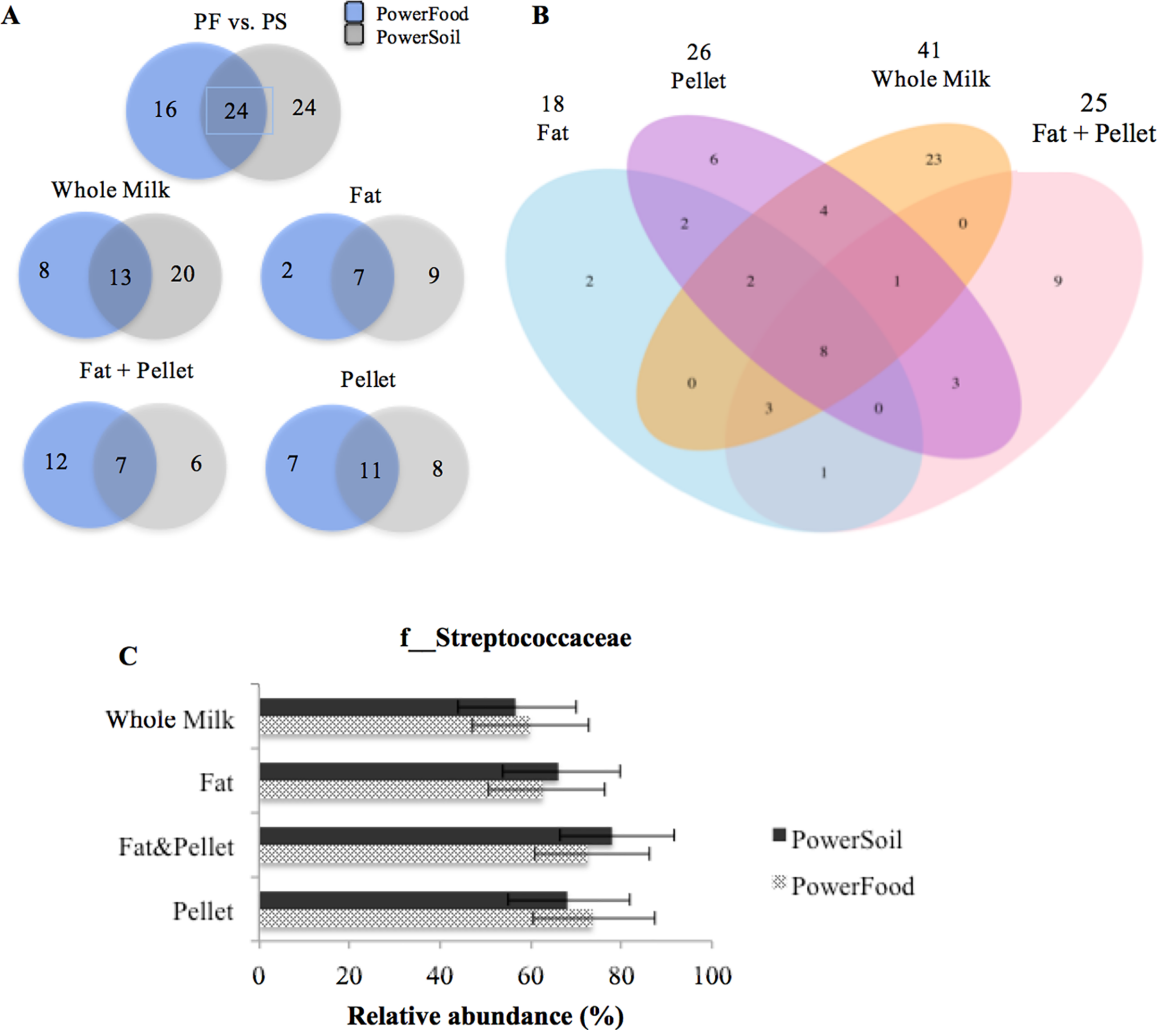
Venn diagram showing the numbers of unique and shared bacterial families among *Streptococcus* spp.-mastitis milk samples according to milk sample fractions: fat, fat + pellet, pellet, and whole milk (WM) and DNA extraction kit (PS and PF) (B). Numbers at the top of the milk sample type name indicate the total number of families detected in the samples processed by that protocol. Mean relative abundance (MRA) of f__Streptococcaceae taxon in each milk sample fractions (C). Error bars represent the standard error of the mean.

Fig 6 shows a Venn diagram of the degree of overlap of bacterial families between the two DNA extraction kits for *Streptococcus* spp.-mastitis milk. Of the 64 families detected, 24 were shared between samples isolated by PowerFood and PowerSoil (Fig 6A). The top five bacterial families identified in *Streptococcus* spp.-mastitis milk samples regardless of the DNA extraction kit and their respective MRA within each DNA extraction kits (PowerFood and PowerSoil respectively) are described as follows: f__Streptococcaceae (60.61% ± 6.7 to 62.60% ± 6.11), f__Enterobacteriaceae (12.91% ± 4.33 to 6.32% ± 2.70), f__Peptostreptococcaceae (6.74% ± 3.34 to 5.52% ± 3.17), f__Ruminococcaceae (2.33% ± 2.22 to 5.01% ± 2.70) and f__Pseudomonadaceae (2.11% ± 2.0 to 4.53% ± 2.54). The number of shared bacterial families within both DNA extraction kits decreased to 13, 7, 7, and 7 when milk sample fractions were included in the analysis (Fig 6A). However, the core shared family between kits holds a MRA of 81.65% (± 3.45). The degree of overlap of bacterial families among the four milk sample fractions is described in Fig 6B. Eight families comprise the core microbiota shared by all four milk sample fractions: f__Streptococcaceae, f__Pseudomonadaceae, f__Ruminococaceae, f__Enterococcaceae, f__Flavobacteriaceae, f__Enterobacteriaceae, f__Brachyspiraceae, and f__Desulfurococcaceae. These eight families combined held a MRA of 81.65% (± 3.4). The description of the shared core and unique families detected in each protocol and their respective relative abundances are given in Tables F and G in S1 File, respectively.

Fig 6C shows that the MRA for all OTUs affiliated to f__Streptococcaceae had good reproducibility (MRA > 50%) across all DNA isolation methods (Fig 6C). All DNA isolation protocols accurately detected f__Streptococcaceae, and no significant differences in the relative abundance of this taxon were observed between DNA isolation procedures (*P*—value > 0.05, Fig 6C).

### Alpha diversity and beta diversity

Since our taxonomic analysis revealed a more varied taxa composition at the phylum and family levels for healthy milk, alpha and beta diversity were calculate and compared between milk fractions and DNA extractions kits within these two milk groups.

Alpha diversity was assessed by measuring Shannon index (quantitative alpha diversity) and Faith’ PD vector (qualitative alpha diversity). No significant differences were observed in either of the alpha diversity measures when milk fraction and kits were compared (Figures B1 and B2 in S1 File). As measured by weighted UniFrac, milk fraction and DNA extraction kits did not affect microbial composition of healthy milk group (Figure B3 in S1 File, PERMANOVA *P*-value = 0.94).

## Discussion

Identification of the most appropriate milk DNA isolation method is especially important for the accurate determination of the mainly bacterial pathogens responsible for the occurrence of udder inflammation. Herein, four distinct milk sample fractions: raw whole milk, milk fat, casein-pellet, and casein-pellet + fat, obtained from milk samples of healthy cows and cows diagnosed with clinical mastitis, were subjected to DNA isolation by beat-beading method to evaluate the best approach for milk microbiota characterization and direct detection of *Klebsiella* spp., *Streptococcus* spp. and *E*. *coli* bacteria. Fourteen percent of samples from healthy milk group were not detected by PCR and consequently not subjected to further amplicon and microbial analysis. These results were expected for healthy milk samples, which typically has very low bacterial load, leading to low DNA concentration [15]. This finding seems to be particular to healthy milk. For instance, in a previous study performed by our research group, DNA extracted from bovine vaginal swab samples from cows within a week before parturition (with no signs of any reproductive disease), resulted in thick bands on gel electrophoresis; even though those samples presented very low bacterial load (quantified by qPCR) and less than 10 colonies in a plate [37]. In this respect, DNA from healthy and mastitis milk that were detected by PCR resulted in adequate amplicon concentrations for next-generation sequencing. Furthermore, microbial DNA isolated from the all four milk sample fractions provided efficiently and uniformly detection of the causative agent of mastitis (*Escherichia coli*, *Klebsiella* spp. and *Streptococcus* spp.).

Generally, assessment of the 16S rRNA amplicon concentration revealed lower amplicon concentration in samples extracted from whole milk than in samples extracted from milk fat, casein-pellet, and casein-pellet + fat, particularly the samples extracted by PowerSoil. The failure of amplification in the PCR assay might be also due to the presence and accumulation of PCR inhibitors after DNA extraction procedure. For instance, Bickely et al (1996), showed that calcium was a major source of PCR inhibition in dairy foods, by competing with magnesium, an essential cofactor of the *Taq*DNA polymerase, whereas fat content seemed to have only minor influence on the amplification efficiency [38]. Additionally, proteinases naturally present in milk (such as milk plasmin) could also inhibit PCR [39]. DNA recovery from distinct milk fractions might also be affected by other important factors, such as centrifugation speed, temperature and time. For example, it has been described that pellet formation of dense particles existent in milk, such as, somatic cells and microbes, is temperature sensitive; in fact, their formation increases with decreasing temperature [40]. Therefore, somatic cells and several bacteria might increase in the fat fractions of milk retrieved by cold agglutination. Additionally, several different centrifugation speeds have been applied to isolate DNA from milk samples [18, 22, 41]. In our study we used a centrifugation speed of 16,000g, and indeed, after centrifugation at high speed (>10,000g), shear forces can also result in breakage of the milk fat globules membranes and some lipid can be deposited in the cell pellet. Although, we have washed the remaining pellet twice with cold PBS, milk lipid might remain attached to the tube when the pellet sample was re-suspended.

Our amplicon-based analysis showed that milk samples obtained from *Streptococcus* spp.*-*mastitis group had higher amplicon concentration when casein-pellet samples were used as substrate. The critical interaction between *Streptococcus* spp. and milk proteins have been shown elsewhere. For instance, Dandoy and colleagues (2011) showed that *Streptococcus thermophiles* biofilm formation in dairy environments depended on the presence of milk proteins and on its ability to hydrolyze casein [42]. Although, some differences in amplicon concentration were observed between milk fractions and DNA extraction methods, all fractions and DNA extraction methods showed great agreement in the taxonomic profile.

One might be concerned about the differences in bead size between the two DNA extraction kits. The larger bead size of PowerSoil beads and the higher temperature in the cell lysis step might have affected its ability to recover bacterial DNA from milk compared to the PowerFood kit, since in overall samples extracted by PowerSoil had lower amplicon concentrations. In addition to the fact that only 27% (28/104) of the families detected in healthy milk were shared among the samples extracted from all four fractions of milk samples; followed by 3% (1/30), 4% (1/23), and 12% (8/64) for the samples from *E*. *coli*-mastitis, *Klebsiella* spp. -mastitis, and *Streptococcus* spp-mastitis, respectively. However, the shared core family among kit and milk fractions held a MRA greater than 85%, regardless of milk-health-status group. Therefore, all DNA extraction protocols showed good reproducibility, with no significant differences in the relative abundance of the main mastitis pathogen taxon across all milk fractions and both DNA extraction kits.

Additionally, when we investigated potential extraction bias for the DNA isolation methods that we tested, alpha diversity and beta diversity of healthy milk group was not affected by milk fraction and DNA extraction kit used; therefore, all milk fractions and extraction methods precisely uncovered the microbial community of healthy milk. In the present study, milk samples from healthy cows were dominated by the Firmicutes and Proteobacteria phyla, and the most abundant bacterial families were represented by f__Ruminococcaceae and f__Enterobacteriaceae. These results are in agreement with previous reports in the literature [27]. Our group has recently measured the diversity within healthy cows’ milk microbiota, however, we formerly found a higher Shannon index in comparison to that observed in the present study [41]. A plausible explanation for this difference between our studies might be the use of different bioinformatics procedures. Ganda et al. (2017) used QIIME 1 pipeline, whereas the present study was analyzed by QIIME 2 pipeline. QIIME 2 contains a new and stricter quality control and OTU assignment tools [33], which might have affected the alpha diversity values herein detected.

Microbial community analysis showed that 16S rRNA sequences detected within the milk samples of the *E*. *coli* and *Klebsiella* spp. mastitis groups were dominated by Proteobacteria. In agreement to the phylum level, analysis at the family level revealed that mastitic milk samples cultured positive for *Escherichia coli* and *Klebsiella* spp. were predominantly affiliated with f__Enterobacteriaceae regardless of the DNA isolation method and milk fractions used. By contrast, sequences from mastitic milk cultured positive for *Streptococcus* spp. were dominated by f__Streptococcacea (distribution ranging from 50% to 80%), followed by the low prevalence of f__Pseudomonadaceae (ranging from 0.12 to 5.5%) and f__Enterococcaceae (ranging from 1 to 5.1%), belonging to the phylum Proteobacteria. This wider distribution f__Streptococcacea within samples from the *Streptococcus* spp.-mastitis samples differed from the scenario seen in the *E*. *coli-*mastitis and *Klebsiela spp*-mastitis, where an almost complete dominance of f__Enterobacteriaceae was detected. These results might be explained by the distinct characteristics of mammary gland infection caused by a coliform, gram-negative bacterium (e.g. *Escherichia coli* and *Klebsiella* spp.) versus a gram-positive bacterium (e.g. *Streptococcus* spp.) [3, 43]. Coliform mastitis typically leads to a more severe and aggressive infection accompanied by a high bacterial growth rate in the mammary gland compared to infections caused by some gram-positive bacteria [43]. Although microbial community analysis revealed that most of the microbial community composition corresponded to milk bacterial species herein tested irrespective of the DNA isolation method, care should be taken when choosing the milk sample type for analysis of milk samples, if the goal of the investigation is to detect the presence of rare bacterial microorganisms.

## Conclusion

All DNA isolation methods and milk fractions tested here recovered DNA suitable for PCR amplification of the 16S rRNA gene from mastitis milk samples. However, the DNA isolation protocols still need to be improved for more efficient isolation of DNA from healthy milk due its typical low bacterial load. Differences in the numbers and types of unique families were observed among the DNA isolation methods regardless of milk-health-status group (healthy and mastitic); however, these were rare families (i.e., low in abundance). The evaluation of relative abundance of main mastitis pathogen taxon across milk fractions and DNA extractions kits showed no significant difference, all milk fractions used for DNA isolation allowed detection of the causative agent of mastitis (*E*. *coli*, *Klebsiella* spp. and *Streptococcus* spp.). Furthermore, only 27% of the families detected in healthy milk were shared among the samples extracted from all fractions of milk, but they held a MRA greater than 80%. Sequences from mastitic milk cultured positive for *Streptococcus* spp. were dominated by f__Streptococcacea, followed by f__Pseudomonadaceae and f__Enterococcaceae in contrast with samples from *E*. *coli*-mastitis and *Klebsiela* spp-mastitis, which were dominated by f__Enterobacteriaceae; fact that can be associated with the intrinsic differences in these pathogens and the mechanisms that they lead to disease.

## Supporting information

S1 File Supplemental 16S data. (DOCX)
