# Supplementary material for: Evaluation of milk sample fractions for characterization of milk microbiota from healthy and clinical mastitis cows
Source: PLoS One. 2018 Mar 21;13(3):e0193671. doi: 10.1371/journal.pone.0193671 (PMC5862444; doi:10.1371/journal.pone.0193671)
Supplement: S1 File — (DOCX) [file pone.0193671.s001.docx]

**Evaluation of milk sample fractions for characterization of milk microbiota from healthy and clinical mastitis cows**

Svetlana Ferreira Lima^1^, Marcela Lucas de Souza Bicalho^1^, Rodrigo Carvalho Bicalho ^1*^

^1^Department of Population Medicine and Diagnostic Sciences, Cornell University, Ithaca, New York, United States of America

^*^Corresponding author: Rodrigo Carvalho Bicalho,

E-mail: [rcb28@cornell.edu](mailto:rcb28@cornell.edu)

**Table A.** Comparison of the number of sequences and operational taxonomic units (OTUs) between milk sample types (whole milk, fat, fat + pellet, and pellet) according to milk-health-status groups (Healthy *E.coli* - mastitis*, Klebsiella* spp. - mastitis, *Streptococcus* spp. - mastitis, and all groups combined) and DNA extraction kits (PorwerFood and PowerSoil). The numbers in parentheses indicate the standard error of the mean. ^a,b,c^ Different superscripts between values indicate a significant difference.

|  |  | **Sequences (n)** | | | | |  | **OTUs (n)** | | | | | |
| --- | --- | --- | --- | --- | --- | --- | --- | --- | --- | --- | --- | --- | --- |
| **Culture groups** |  | WM^1^ | Fat | F+P^2^ | Pellet | *P*-value |  | WM | Fat | F+P | Pellet | *P*-value |  |
| **PowerFood** |  |  |  |  |  |  |  |  |  |  |  |  |  |
| *Healthy* |  | 92,449.8 (14,183.6) | 96,982.9 (15,044.0) | 62,067.4 (12,829.5) | 78,636.7 (12,283.3) | 0.27 |  | 55,707.9 (12,236.1) | 48,676.4 (10.857.0) | 32,947 (9,258.9) | 45,156.2 (8,864.7) | 0.69 |  |
| *Escherichia coli* |  | 77,462.0 (19,135.1) | 85,728.2 (19135.1) | 68,356.2 (19,135.1) | 81,823.0 (22,095.3) | 0.92 |  | 24,221.5 (3,907.0) | 27,238.5 (3,907.0) | 18,870.7 (3,907.0) | 25,868.7 (4,511.4) | 0.48 |  |
| *Klebsiella* spp. |  | 72,259.7 (21,875.0) | 48,780.5 (21,875.0) | 61657.75 (21,875.0) | 72,935.5 (21,875.0) | 0.84 |  | 3,1317.2 (4,932.9) | 21,695.2 (4,932.9) | 20,274.2 (4,932.9) | 27,522.0 (4,932.9) | 0.39 |  |
| *Streptococcus* spp. |  | 55,075.1 (9,714.1) | 44,058.2 (10,560.3) | 71,069.6 (10,560.3 | 67,644.8 (11,075.7) | 0.26 |  | 27,684.1 (6,6602.2) | 23,120.0 (7,177.3) | 39769.0 (7,177.3) | 30,626.4 (7,527.6) | 0.41 |  |
| Total |  | 61,843 (7,130.2) | 69,634 (7,338.7) | 61,897 (7,313.9) | 68,246 (7,124.6) | 0.71 |  | 3,4409 (4,630.0) | 26,427 (4,759.7) | 29,212 (4,620.7) | 31,215 (4,673.4) | 0.64 |  |
| **PowerSoil** |  |  |  |  |  |  |  |  |  |  |  |  |  |
| *Healthy* |  | 69,840.6 (13,917.7) | 64,387.4 (15,560.5) | 72,775.2 (13,917.7) | 63,337.4 (13,917.7) | 0.95 |  | 41,940.2 (9,505.9) | 39,374.1 (10,627.9) | 26,808.4 (9,505.9) | 37,011.8 (9,505.9) | 0.86 |  |
| *Escherichia coli* |  | 81,417 (23,453.6) | 89,387.5 (20,311.4) | 63,141.7 (23,453.6) | 79,525.7 (20,311.4) | 0.86 |  | 26,271.7 (8,865.7) | 30,421.2 (7.677.9) | 27,526.7 (8,865.7) | 31,977.2 (7,677.9) | 0.95 |  |
| *Klebsiella* spp. |  | 67,363.5 (21,558.8) | 81,043.2 (21,558.8) | 60,895.5 (21,558.8) | 74,181.2 (21,558.8) | 0.91 |  | 24,998.2 (3,777.8) | 33,842.2 (3,777.8) | 24,944.7 (3,777.9) | 24,489.2 (3,777.9) | 0.28 |  |
| *Streptococcus* spp. |  | 47,555.1 (7,773.5) | 56,480.1 (7,773.5) | 56,752.1 (8,863.1) | 71,954.6 (8,450.6) | 0.22 |  | 24,126.6 (3,772.0) | 22,172.5 (3,772.0) | 34,084.4 (4,300.7) | 27,362.5 (4,100.6) | 0.19 |  |
| Total |  | 73,893 (7,618.7) | 63,216 (7,832.1) | 67,035 (7,603.3) | 72,799 (7,690.0) | 0.79 |  | 28,950 (3,853.1) | 29,647 (3,965.7) | 26,587 (3,952.4) | 28,149 (3,850.1) | 0.94 |  |
| **All groups combined** |  | 74,121 (7,014.3) | 68,801 (7,349.6) | 75,602 (7,007.0) | 67,594 (7,158.7) | 0.77 |  | 63,680 (7,051.2) | 71,322 (7,247.7) | 65,704 (7,384.2) | 72,008 (7,113.0) | 0.86 |  |

WM^1^: whole milk; F+P^2^: fat + pellet

**Table B.** Description of the core shared microbiota, characterized as the bacterial families detected in all milk fractions (whole milk, fat, fat + pellet, and pellet) of healthy milk samples.

|  | **Mean relative abundance (Standard Error)** | | | |  |
| --- | --- | --- | --- | --- | --- |
| **Family** | Whole Milk | Fat | Fat + Pellet | Pellet | FDR *P*-value |
| f__Ruminococcaceae | 32.9 (7.9) | 16.6 (7.1) | 22.4 (7.3) | 13.2 (5.8) | 0.71 |
| f__Enterobacteriaceae | 13.1 (7.1) | 12.4 (6.4) | 6.4 (4.2) | 12.9 (6.6) | 0.55 |
| f__Bacillaceae | 11.2 (4.7) | 9.2 (6.0) | 11.3 (5.5) | 1.7 (0.7) | 0.85 |
| f__Pseudomonadaceae | 5.7 (5.7) | 6.5 (6.4) | 5.3 (5.3) | 5.0 (5.0) | 0.82 |
| f__Moraxellaceae | 5.3 (3.8) | 1.3 (0.4) | 4.6 (2.7) | 2.7 (2.4) | 0.77 |
| f__Streptococcaceae | 4.9 (4.6) | 16.2 (9.0) | 1.4 (1.2) | 5.3 (4.0) | 0.73 |
| f__Rhodobacteraceae | 4.9 (4.7) | 0.02 (0.02) | 0.2 (0.1) | 0.2 (0.1) | 0.68 |
| f__Lachnospiraceae | 2.5 (0.8) | 0.7 (0.5) | 1.9 (0.7) | 1.4 (0.05) | 0.79 |
| f__Corynebacteriaceae | 1.4 (0.9) | 0.2 (0.2) | 0.2 (0.2) | 7.9 (4.8) | 0.86 |
| f__Bacteroidaceae | 1.3 (0.4) | 0.7 (0.7) | 1.5 (0.6) | 1.3 (0.4) | 0.88 |
| f__Clostridiaceae | 0.8 (0.3) | 0.8 (0.4) | 0.2 (0.1) | 0.1 (0.07) | 0.78 |
| f__Oxalobacteraceae | 0.7 (0.4) | 1.6 (1.0) | 0.5 (0.5) | 0.2 (0.2) | 0.74 |
| f__Paraprevotellaceae | 0.6 (0.4) | 0.01 (0.01) | 0.3 (0.1) | 0.1 (0.1) | 0.89 |
| f__Spirochaetaceae | 0.6 (0.2) | 6.3 (6.0) | 0.2 (0.1) | 0.1 (0.05) | 0.78 |
| f__Alcaligenaceae | 0.6 (0.4) | 0.1 (0.1) | 0.2 (0.2) | 0.3 (0.2) | 0.78 |
| f__Staphylococcaceae | 0.5 (0.34) | 13.9 (9.2) | 18.3 (8.5) | 24.1 (8.7) | 0.81 |
| f__Aerococcaceae | 0.4 (0.4) | 0.2 (0.2) | 0.7 (0.4) | 0.9 (0.6) | 0.80 |
| f__Sphingobacteriaceae | 0.4 (0.3) | 0.6 (0.4) | 0.2 (0.2) | 0.4 (0.2) | 0.89 |
| f__Porphyromonadaceae | 0.4 (0.2) | 0.1 (0.07) | 0.4 (0.3) | 0.3 (0.2) | 0.73 |
| f__Prevotellaceae | 0.3 (0.1) | 0.1 (0.09) | 0.4 (0.2) | 0.1 (0.07) | 0.66 |
| f__Bifidobacteriaceae | 0.2 (0.07) | 0.02 (0.02) | 0.2 (0.1) | 0.1 (0.05) | 0.76 |
| f__Tissierellaceae | 0.1 (0.1) | 0.3 (0.2) | 0.1 (0.1) | 0.05 (0.05) | 0.66 |
| f__Peptostreptococcaceae | 0.1 (0.1) | 0.3 (0.1) | 6.3 (4.0) | 3.6 (3.3) | 0.75 |
| f__Phyllobacteriaceae | 0.1 (0.1) | 0.2 (0.1) | 0.7 (0.5) | 0.2 (0.1) | 0.90 |
| f__Veillonellaceae | 0.1 (0.1) | 0.1 (0.1) | 0.1 (0.07) | 0.2 (0.2) | 0.91 |
| f__Mogibacteriaceae | 0.1 (0.04) | 0.07 (0.06) | 0.2 (0.1) | 0.007 (0.007) | 0.68 |
| f__Flavobacteriaceae | 0.09 (0.08) | 0.3 (0.3) | 3.6 (0.6) | 0.2 (0.1) | 0.98 |
| f__Dermabacteraceae | 0.06 (0.06) | 0.3 (0.3) | 1.3 (1.1) | 3.0 (2.3) | 0.71 |

**Table C.** Description of the unique OTUs detected in healthy milk samples extracted from whole milk, fat, fat + pellet, and pellet.

|  | **Mean Relative Abundance (Standard Error)** | | | |
| --- | --- | --- | --- | --- |
| **Family** | Whole Milk | Fat | Fat + Pellet | Pellet |
| f__Gemellaceae | 0.6 (0.6) | 0 | 0 | 0 |
| f__Gordoniaceae | 0.4 (0.4) | 0 | 0 | 0 |
| f__Deinococcaceae | 0.4 (0.4) | 0 | 0 | 0 |
| f__Methylococcaceae | 0.3 (0.3) | 0 | 0 | 0 |
| f__Campylobacteraceae | 0.2 (0.1) | 0 | 0 | 0 |
| f__Aeromonadaceae | 0.2 (0.1) | 0 | 0 | 0 |
| f__Brachyspiraceae | 0.1 (0.1) | 0 | 0 | 0 |
| f__Sphingomonadaceae | 0.08 (0.06) | 0 | 0 | 0 |
| f__Marinilabiaceae | 0.04 (0.04) | 0 | 0 | 0 |
| f__Chthoniobacteraceae | 0.03 (0.03) | 0 | 0 | 0 |
| f__Verrucomicrobiaceae | 0.03 (0.02) | 0 | 0 | 0 |
| f__Halomonadaceae | 0.02 (0.020 | 0 | 0 | 0 |
| f__Erysipelotrichaceae | 0.02 (0.01) | 0 | 0 | 0 |
| f__Fibrobacteraceae | 0.01 (0.01) | 0 | 0 | 0 |
| f__RFP12 | 0.01 (0.01) | 0 | 0 | 0 |
| f__Thermoanaerobacteraceae | 0.01 (0.01) | 0 | 0 | 0 |
| f__Thermoactinomycetaceae | 0.008 (0.008) | 0 | 0 | 0 |
| f__Planctomycetaceae | 0.007 (0.007) | 0 | 0 | 0 |
| f__Halobacteriaceae | 0 | 3.6 (3.6) | 0 | 0 |
| f__Leptotrichiaceae | 0 | 3.4 (3.4) | 0 | 0 |
| f__Rhizobiaceae | 0 | 0.07 (0.07) | 0 | 0 |
| f__Hyphomicrobiaceae | 0 | 0 | 1.1 (1.0) | 0 |
| f__Nocardiopsaceae | 0 | 0 | 0.2 (0.2) | 0 |
| f__Hyphomonadaceae | 0 | 0 | 0.1 (0.1) | 0 |
| f__Micromonosporaceae | 0 | 0 | 0.1 (0.1) | 0 |
| f__Dehalobacteriaceae | 0 | 0 | 0.05 (0.04) | 0 |
| f__Anaeroplasmataceae | 0 | 0 | 0.04 (0.02) | 0 |
| f__Psychromonadaceae | 0 | 0 | 0.02 (0.01) | 0 |
| f__Nocardiaceae | 0 | 0 | 0 | 0.4 (0.2) |
| f__Glycomycetaceae | 0 | 0 | 0 | 0.06 (0.04) |
| f__Rhabdochlamydiaceae | 0 | 0 | 0 | 0.02 (0.02) |
| f__Thermobaculaceae | 0 | 0 | 0 | 0.01 (0.01) |

**Table D.** Description of the unique OTUs detected in *Escherchia coli*-mastitis milk extracted from whole milk, fat, fat + pellet, and pellet.

|  | **Mean Relative Abundance (Standard Error)** | | | |
| --- | --- | --- | --- | --- |
| **Family** | Whole Milk | Fat | Fat + Pellet | Pellet |
| f__Streptococcaceae | 0.9 (0.9) | 0 | 0 | 0 |
| f__Bacillaceae | 0.6 (0.6) | 0 | 0 | 0 |
| f__Micrococcaceae | 0.1 (0.1) | 0 | 0 | 0 |
| f__Spirochaetaceae | 0.02 (0.02) | 0 | 0 | 0 |
| f__Anaerolinaceae | 0 | 0.9 (0.9) | 0 | 0 |
| f__Chthoniobacteraceae | 0 | 0.6 (0.6) | 0 | 0 |
| f__Xanthomonadaceae | 0 | 0.5 (0.5) | 0 | 0 |
| f__Leptotrichiaceae | 0 | 0.5 (0.5) | 0 | 0 |
| f__Turicibacteraceae | 0 | 0.2 (0.2) | 0 | 0 |
| f__Clostridiaceae | 0 | 0.1 (0.1) | 0 | 0 |
| f__RFP12 | 0 | 0.1 (0.1) | 0 | 0 |
| f__Sphingomonadaceae | 0 | 0.04 (0.04) | 0 | 0 |
| f__Halobacteriaceae | 0 | 0.03 (0.03) | 0 | 0 |
| f__Flammeovirgaceae | 0 | 0.01 (0.01) | 0 | 0 |
| f__Ulvophyceae | 0 | 0 | 0.9 (0.9) | 0 |
| f__Peptostreptococcaceae | 0 | 0 | 0 | 2.3 (2.3) |
| f__Bacteroidaceae | 0 | 0 | 0 | 1.3 (0.8) |
| f__Moraxellaceae | 0 | 0 | 0 | 0.5 (0.5) |
| f__Pirellulaceae | 0 | 0 | 0 | 0.3 (0.5) |
| f__S24-7 | 0 | 0 | 0 | 0.2 (0.2) |
| f__Aerococcaceae | 0 | 0 | 0 | 0.2 (0.2) |
| f__Veillonellaceae | 0 | 0 | 0 | 0.1 (0.1) |
| f__Prevotellaceae | 0 | 0 | 0 | 0.1 (0.1) |
| f__Dermacoccaceae | 0 | 0 | 0 | 0.1 (0.1) |
| f__Paraprevotellaceae | 0 | 0 | 0 | 0.05 (0.05) |
| f__Flavobacteriaceae | 0 | 0 | 0 | 0.05 (0.05) |
| f__Porphyromonadaceae | 0 | 0 | 0 | 0.05 (0.05) |

**Table E.** Description of the unique OTUs detected in *Klebsiella* spp-mastitis milk extracted from whole milk, fat, fat + pellet, and pellet.

|  | **Mean Relative Abundance (Standard Error)** | | | |
| --- | --- | --- | --- | --- |
| **Family** | Whole Milk | Fat | Fat + Pellet | Pellet |
| f__Peptostreptococcaceae | 2.5 (2.5) | 0 | 0 | 0 |
| f__Fusobacteriaceae | 1.2 (1.2) | 0 | 0 | 0 |
| f__Lachnospiraceae | 0.05 (0.05) | 0 | 0 | 0 |
| f__S24-7 | 0.04 (0.04) | 0 | 0 | 0 |
| f__Spirochaetaceae | 0.04 (0.04) | 0 | 0 | 0 |
| f__Caulobacteraceae | 0.03 (0.03) | 0 | 0 | 0 |
| f__Xanthomonadaceae | 0.03 (0.03) | 0 | 0 | 0 |
| f__Bacillaceae | 0 | 0.4 (0.4) | 0 | 0 |
| f__Porphyromonadaceae | 0 | 0.03 (0.3) | 0 | 0 |
| f__Thermaceae | 0 | 0.001 (0.001) | 0 | 0 |
| f__Desulfurococcaceae | 0 | 0 | 0.04 (0.04) | 0 |
| f__Anaerolinaceae | 0 | 0 | 0 | 0.3 (0.3) |
| f__Chlamydomonadaceae | 0 | 0 | 0 | 0.04 (0.04) |
| f__Prevotellaceae | 0 | 0 | 0 | 0.03 (0.03) |
| f__Pasteurellaceae | 0 | 0 | 0 | 0.001580675 |

**Table F.** Description of the core shared microbiota, characterized as the bacterial families detected in all milk fractions (whole milk, fat, fat + pellet, and pellet) of *Streptococcus* spp.-mastitis.

|  | **Mean Relative Abundance (Standard Error)** | | | |  |
| --- | --- | --- | --- | --- | --- |
| **Family** | **Whole Milk** | **Fat** | **Fat&Pellet** | **Pellet** | **FDR**  ***P*-value** |
| f__Streptococcaceae | 53.7 (8.8) | 59.1 (9.0) | 71.7 (9.0) | 64.3 (9.4) | 0.6 |
| f__Pseudomonadaceae | 4.0 (3.4) | 5.5 (4.3) | 0.12 (0.08) | 3.3 (2.0) | 0.9 |
| f__Enterococcaceae | 2.4 (2.4) | 5.1 (3.61) | 1.0 (1.0) | 4.8 (4.8) | 0.9 |
| f__Ruminococcaceae | 4.4 (3.1) | 4.3 (4.2) | 4.9 (4.7) | 1.0 (0.6) | 0.7 |
| f__Flavobacteriaceae | 3.6 (2.3) | 4.0 (4.0) | 0.6 (0.3) | 0.2 (0.2) | 0.8 |
| f__Enterobacteriaceae | 8.6 (4.0) | 10.9 (5.8) | 6.1 (4.5) | 12.6 (6.0) | 0.7 |
| f__Brachyspiraceae | 0.8 (0.8) | 0.4 (0.4) | 0.3 (0.3) | 1.8 (1.8) | 1.0 |
| f__Desulfurococcaceae | 0.4 (0.4) | 0.3 (0.3) | 2.5 (2.5) | 1.7 (1.2) | 0.6 |

**Table G.** Description of the unique OTUs detected in the microbial communities extracted from whole milk, fat, fat + pellet, and pellet of *Streptococcus* spp.-mastitis.

|  | **Mean Relative Abundance (Standard Error)** | | | |
| --- | --- | --- | --- | --- |
| **Family** | Whole Milk | Fat | Fat + Pellet | Pellet |
| f__Oxalobacteraceae | 3.7 (3.7) | 0 | 0 | 0 |
| f__Bacillaceae | 3.7 (3.0) | 0 | 0 | 0 |
| f__Aerococcaceae | 1.1 (1.1) | 0 | 0 | 0 |
| f__Gordoniaceae | 0.6 (0.5) | 0 | 0 | 0 |
| f__Halobacteroidaceae | 0.4 (0.4) | 0 | 0 | 0 |
| f__Ulvophyceae | 0.4 (0.4) | 0 | 0 | 0 |
| f__Caldicellulosiruptoraceae | 0.4 (0.3) | 0 | 0 | 0 |
| f__Fusobacteriaceae | 0.3 (0.3) | 0 | 0 | 0 |
| f__Deinococcaceae | 0.2 (0.2) | 0 | 0 | 0 |
| f__Bogoriellaceae | 0.2 (0.2) | 0 | 0 | 0 |
| f__Nocardioidaceae | 0.2 (0.2) | 0 | 0 | 0 |
| f__Pseudonocardiaceae | 0.2 (0.2) | 0 | 0 | 0 |
| f__Oceanospirillaceae | 0.08 (0.8) | 0 | 0 | 0 |
| f__Phyllobacteriaceae | 0.07 (0.07) | 0 | 0 | 0 |
| f__Staphylococcaceae | 0.07 (0.07) | 0 | 0 | 0 |
| f__Xenococcaceae | 0.06 (0.06) | 0 | 0 | 0 |
| f__Chitinophagaceae | 0.03 (0.03) | 0 | 0 | 0 |
| f__Erythrobacteraceae | 0.03 (0.02) | 0 | 0 | 0 |
| f__Pseudanabaenaceae | 0.02 (0.02) | 0 | 0 | 0 |
| f__Streptomycetaceae | 0.02 (0.02) | 0 | 0 | 0 |
| f__Geodermatophilaceae | 0.01 (0.01) | 0 | 0 | 0 |
| f__Sphingomonadaceae | 0.01 (0.01) | 0 | 0 | 0 |
| f__Tissierellaceae | 0.01 (0.01) | 0 | 0 | 0 |
| f__Pasteurellaceae | 0 | 0.1 (0.1) | 0 | 0 |
| f__Halomonadaceae | 0 | 0.002 (0.002) | 0 | 0 |
| f__Sinobacteraceae | 0 | 0 | 0.07 (0.074) | 0 |
| f__Bradyrhizobiaceae | 0 | 0 | 0.06 (0.06) | 0 |
| f__Leptotrichiaceae | 0 | 0 | 0.06 (0.06) | 0 |
| f__Mycoplasmataceae | 0 | 0 | 0.06 (0.06) | 0 |
| f__Gemmataceae | 0 | 0 | 0.02 (0.02) | 0 |
| f__Helicobacteraceae | 0 | 0 | 0.02 (0.02) | 0 |
| f__Thermoanaerobacteraceae | 0 | 0 | 0.005 (0.005) | 0 |
| f__Desulfohalobiaceae | 0 | 0 | 0.004 (0.004) | 0 |
| f__Mogibacteriaceae | 0 | 0 | 0.002 (0.002) | 0 |
| f__Comamonadaceae | 0 | 0 | 0 | 2.0 (2.0) |
| f__Rhodobacteraceae | 0 | 0 | 0 | 0.5 (0.5) |
| f__Corynebacteriaceae | 0 | 0 | 0 | 0.1 (0.1) |
| f__Veillonellaceae | 0 | 0 | 0 | 0.02 (0.02) |
| f__Prevotellaceae | 0 | 0 | 0 | 0.01 (0.01) |
| f__S24-7 | 0 | 0 | 0 | 0.01 (0.01) |

**Fig A**. Mean relative abundance (MRA) of Firmicutes and Proteobacteria phyla in healthy milk group (A1 and A2, respectively) according to milk fractions and DNA extraction kits. Kruskal-Wallis test followed by Benjamini-Hochberg false discovery rate (FDR) were used to generate the corrected *P*-values. Error bars represent the standard error of the mean.


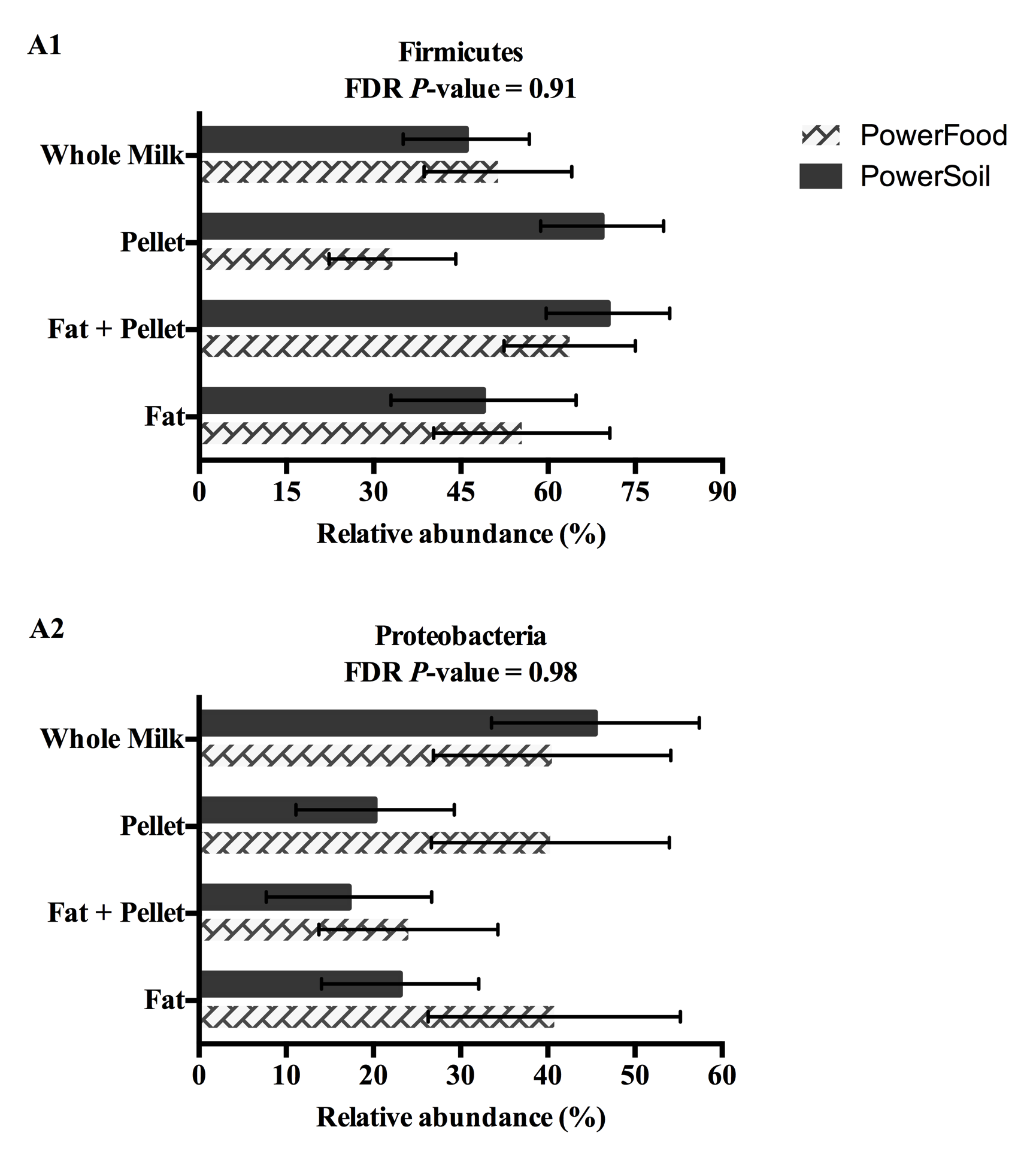


**Fig B.** Alpha diversity represented by Shannon index (B1) and Faith’s Phylogenetic Vector (B2) of healthy milk group according to milk sample fractions and DNA extraction kits. Error bars represent the standard error of the mean. Principal coordinating analysis (PCoA) in the context of Weighted UniFrac distance matrix of healthy milk group according to milk sample fraction and DNA extraction kits (B3). *P* - value listed for differential clustering assessed by PERMANOVA test (B3).


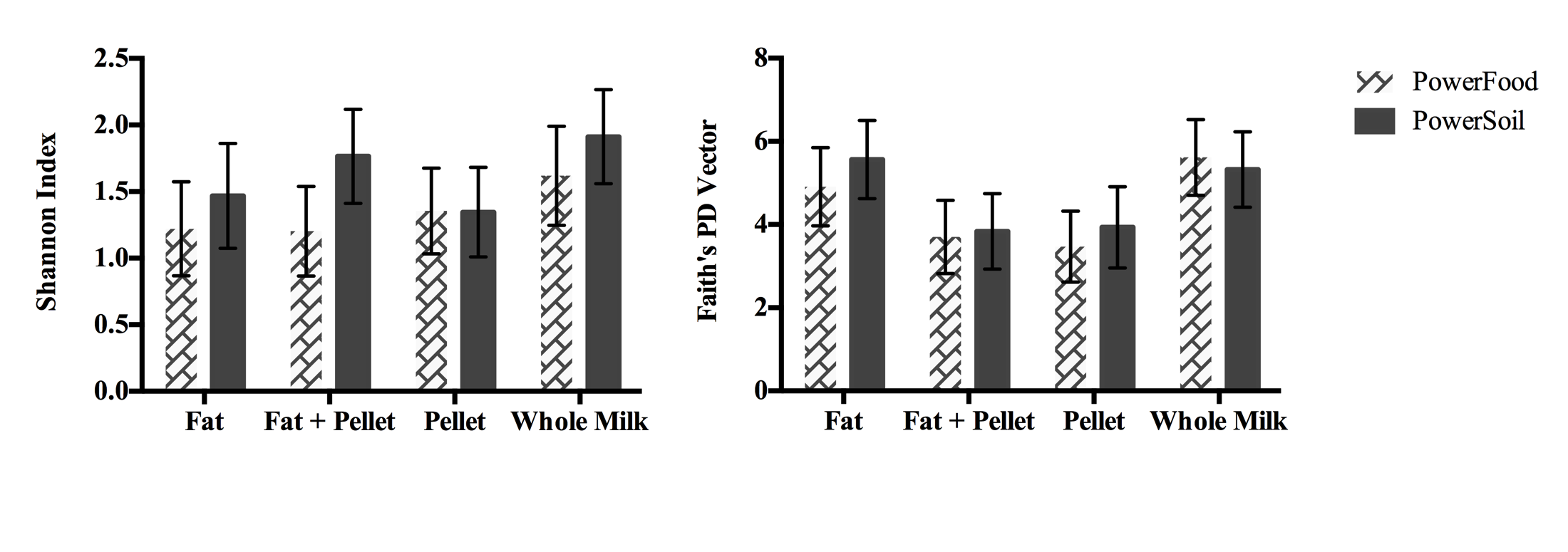


**B1
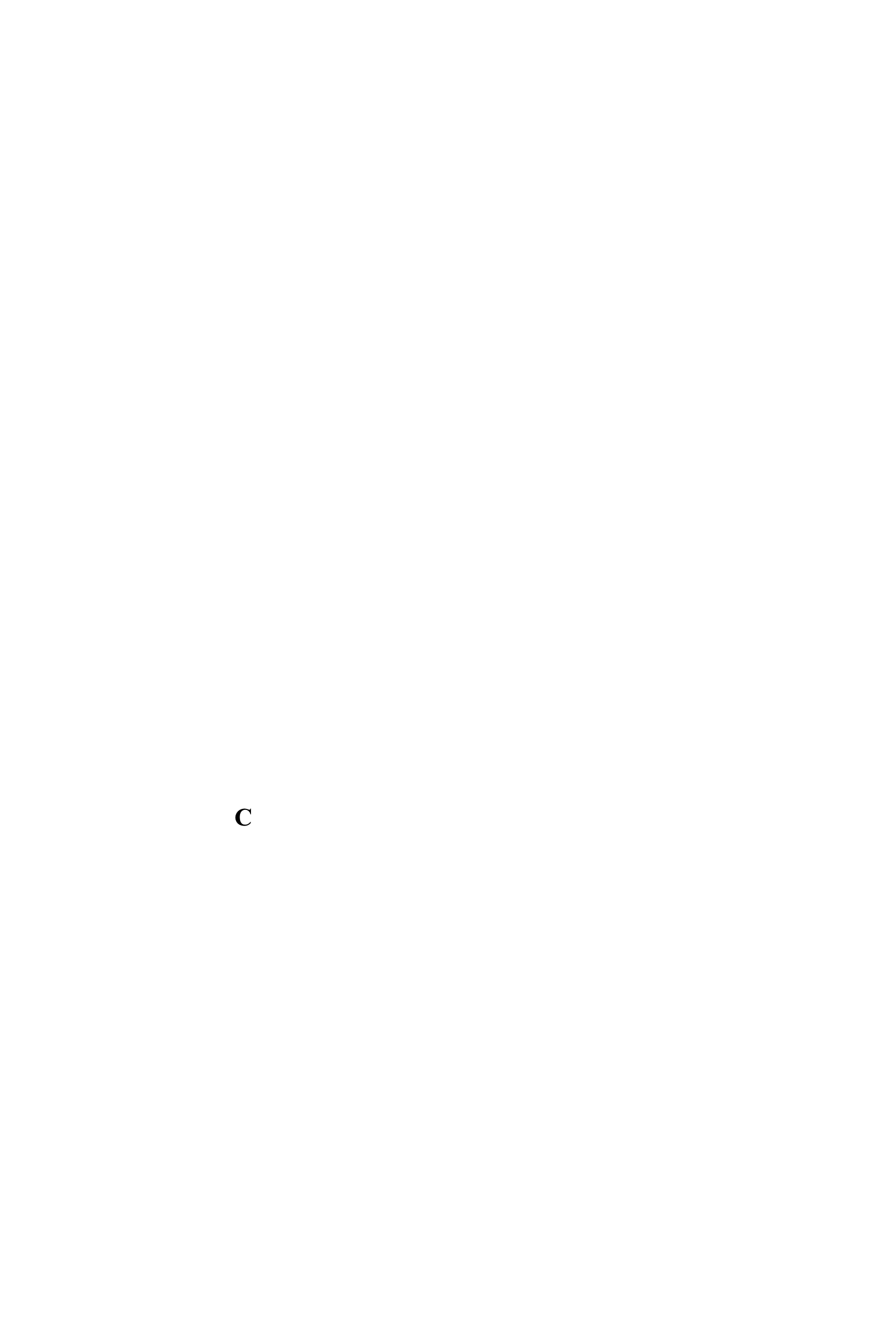
**

**B2**


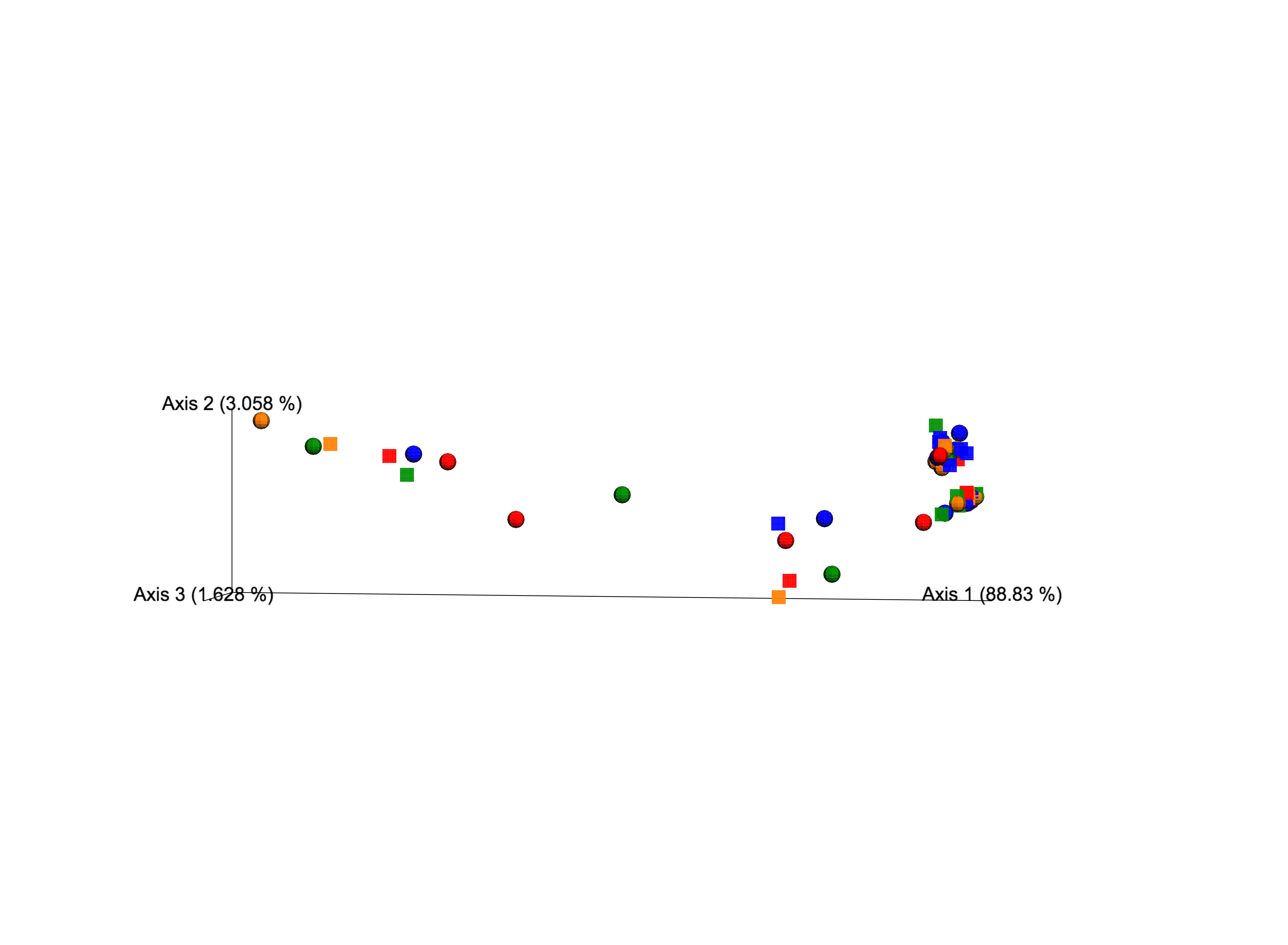


**B3**

Pellet

WM

Fat

Fat + Pellet

**Protocol**

PowerFood

PowerSoil

**Kit**

PERMANOVA

*P*-value = 0.94
